# Bayesian estimation of directed functional coupling from brain recordings

**DOI:** 10.1371/journal.pone.0177359

**Published:** 2017-05-18

**Authors:** Danilo Benozzo, Pasi Jylänki, Emanuele Olivetti, Paolo Avesani, Marcel A. J. van Gerven

**Affiliations:** 1 NeuroInformatics Laboratory (NILab), Bruno Kessler Foundation, Trento, Italy; 2 Information Engineering and Computer Science Department (DISI), University of Trento, Trento, Italy; 3 Center for Mind and Brain Sciences (CIMeC), University of Trento, Trento, Italy; 4 Radboud University, Donders Institute for Brain, Cognition and Behaviour, Nijmegen, the Netherlands; University of Rijeka, CROATIA

## Abstract

In many fields of science, there is the need of assessing the causal influences among time series. Especially in neuroscience, understanding the causal interactions between brain regions is of primary importance. A family of measures have been developed from the parametric implementation of the Granger criteria of causality based on the linear autoregressive modelling of the signals. We propose a new Bayesian method for linear model identification with a structured prior (GMEP) aiming to apply it as linear regression method in the context of the parametric Granger causal inference. GMEP assumes a Gaussian scale mixture distribution for the group sparsity prior and it enables flexible definition of the coefficient groups. Approximate posterior inference is achieved using Expectation Propagation for both the linear coefficients and the hyperparameters. GMEP is investigated both on simulated data and on empirical fMRI data in which we show how adding information on the sparsity structure of the coefficients positively improves the inference process. In the same simulation framework, GMEP is compared with others standard linear regression methods. Moreover, the causal inferences derived from GMEP estimates and from a standard Granger method are compared across simulated datasets of different dimensionality, density connection and level of noise. GMEP allows a better model identification and consequent causal inference when prior knowledge on the sparsity structure are integrated in the structured prior.

## Introduction

Wiener-Granger causality is a well-established approach to study causality between time series [1]. This approach is based on the definition of causality proposed by Wiener [2] which considers one time series the cause of another if the latter is better predicted by including information about the first. An implementation of this concept was proposed by Granger [3] who used it to estimate causality between stochastic processes, modelling them as linear autoregressive (AR) models. Specifically, the parametric implementation of Granger causality (GC) identifies a causal interaction between two time series by first modelling them through an AR model and then by comparing how the prediction error changes if each time series is modelled just using its own past values or also including the past values of the others.

Granger causality has been applied in many different fields [4–7] and it has become a popular method for identifying causal interactions due to its simplicity and intuitive meaning. This holds particularly in neuroscience, where the understanding of causal interactions among brain areas is of primary importance. According to the terminology adopted in neuroscience, the Wiener-Granger method belongs to the group of the directed functional connectivity methods [8] since it aims to identify the direction of the statistical dependences among a set of brain signals, without making any assumptions about the mechanistic nature of these connections. In neuroscientific applications, given the concurrent acquisition of time series from different brain regions, the problem of inferring causal interactions should take into account the multivariate nature of the data. This desideratum was considered in [9, 10], where a generalization of GC was proposed that relies on the multivariate autoregressive (MAR) model, thereby moving beyond pairwise causal interactions. Apart from the well-known Granger method, several others solutions have been developed. The vast majority of them still starts from the Wiener idea of causality and from modelling the causal interactions through an MAR model [11, 12].

All the approaches that involve MAR modelling require the estimation of the model coefficients as well as of the residual covariance matrix. Since each time point is modelled through a multivariate linear model, this estimation procedure can be shown to be equivalent to solving a multivariate linear regression problem. Due to the nature of neuroscientific datasets, the number of coefficients can be massive. This occurs because signals are acquired from a large number of brain areas. These areas are expressed in term of single or groups of voxels in the fMRI case, and sensors or sources in the MEG/EEG case. Defining *d*_*y*_ as the number of time series and *p* the so-called order of the MAR model that indicates how many time lags are involved in the modelling of the present time point, the total number of MAR coefficients is *p* × *d*_*y*_ × *d*_*y*_. Hence, the number of unknown coefficients is more than quadratic with respect to the number of time series. This point reveals a crucial property of the multivariate linear regression problem since it is a bottleneck for the scalability of most of the standard linear regression techniques.

The simplest linear regression method is the ordinary least squares (OLS) method. OLS computes the solution by minimizing the root mean square error. There are many examples in which this approach, or variations of it, are considered in the literature [1, 11, 13–15]. As mentioned in [16], overfitting is the main risk of OLS when a large number of independent variables are used in the modelling. Even more problematic is the regression if the number of independent variables exceeds the number of observations since the least squares solution will not be unique. Moreover, the high correlation between neural time series provides an additional challenge to OLS estimators [17]. In order to overcome the limitations of OLS, one may attempt to regularize the solution [18]. Regularization is done by including in the argument of the cost function a term that controls the overall amplitude of the estimates. This term is generally called penalty term and the resulting approach, penalized regression model. In [19] the authors analysed the use of different penalized regression models, including the well known ridge regression and lasso, for directed functional connectivity estimation. In [18] the elastic net regularizer was considered. Elastic net considers both the penalty terms of ridge and lasso, thus both *l*_1_ and *l*_2_ norms of the coefficients are linearly combined in the cost function. A more sophisticated version of the standard penalized regression models, named group lasso, was proposed in [20] where the authors introduced the concept of grouped variables. By grouped variables, it is meant that the independent variables are clustered in order to find important explanatory variables in predicting the dependent variable. The clustering of the independent variables implies a related clustering of the coefficients at which a separate penalty term is associated. This allows each cluster of coefficients to be separately regularized instead of a global regularization of the whole coefficient vector. This family of methods is often referred to as group sparse regularisation methods. In [21], the asymptotic properties of group lasso were analysed in terms of consistency, normality and uniqueness of the estimate. While in [22], a comparison between standard lasso and group lasso is presented by focusing on the conditions under which group lasso outperforms lasso. In the context of causal inference in multivariate time series, group lasso was studied and compared with non-grouped penalized regression models in [23]. In that work, group lasso was used to enforce coefficient sparsity by grouping together the coefficients connecting the same pair of signals across all time lags. An example application of group lasso with pseudo-EEG data is discussed in [16].

In the Bayesian setting, regularization can be interpreted as imposing a particular prior on the model coefficients. As pointed out in [24], major advantages of Bayesian inference are: the possibility to include prior knowledge in the model definition, the use of model evidence as a measure to compare hypotheses, and finally a quantification of residual uncertainty as captured by the posterior distribution. Several Bayesian approaches were presented in the literature for group sparse modelling, in which the idea of structured priors is exploited to enforce sparsity on the coefficients. The concept of structured (or group sparsity or sparsity-enforcing) priors in the Bayesian setting conveys the same idea of grouped variables. Thus, a structured prior refers to a clustering of the coefficients in which elements in the same group are drawn from the same prior distribution. In [25] a multivariate Gaussian prior was assumed for each group and the expectation maximization (EM) algorithm was used for the inference. A similar approach is presented in [26] where a Dirichlet process prior was employed as structured prior while variational Bayesian was used for the estimate. Other examples have been developed in [27–29]. Regarding the application of group sparsity promoting methods in the context of neuroscience, we mention the approach proposed in [30]. In that case, a multidimensional Gaussian distribution was associated to the structured prior and the inference was done in the variational Bayesian framework. This approach was also used in [31]. Another example of a sparse Bayesian regression method is that of [17]. Here, the authors assume that the coefficients are spatially smooth within each time lag and a closed-form solution is obtained by using conjugate priors. The spike-and-slab distribution represents yet another way to constrain the amplitude of the coefficients. This distribution is investigated in [32, 33] as sparsity-enforcing prior for linear regression.

Here, we propose a novel approach for Bayesian group sparse modelling, called GMEP. The source code is available at https://github.com/ccnlab/GMEP. The name GMEP refers to the Gaussian scale Mixture distribution that is adopted to form a general class of group sparsity priors, and to the Expectation Propagation framework that is used as an efficient method for approximate Bayesian inference. The model is formulated in a general way that enables flexible definition of various non-conjugate observation models. Furthermore, structured priors can be specified using hyperparameters that themselves rely on a multivariate Gaussian prior. The hierarchical structure of the model allows the priors and the hyperparameter vector not to be fixed but modelled by the chosen prior distributions. The posterior is approximated using EP [34] for both the linear coefficients and the hyperparameters. EP has shown to be very accurate and reasonably fast with respect to variational Bayes and Markov chian Monte Carlo [35]. A drawback of EP is the no guarantee of convergence but if properly implemented, convergence can be reliably reached [36].

In this paper, we use GMEP as the basis for a linear regression model to identify a MAR model and to infer the connectivity structure of a given sample of time series. The resulting approach is evaluated both on simulated and empirical fMRI data. The analysis on the simulated dataset aims firstly to compare GMEP with the most commonly used linear regression methods for MAR estimation. Then our approach is evaluated under different prior definitions that represent different sparsity structures of the coefficients. Moreover, we compare the predictive capability of GMEP, and of the multivariate Granger Causality toolbox (MVGC) [15], across different noise levels. Finally, the experiments conducted on the empirical fMRI dataset are meant to investigate the plausibility of some hypotheses related to the sparsity structure of the MAR coefficients. The most realistic hypothesis among the considered ones, is chosen to estimate the directed functional structure in the fMRI time series.

## Methods

In this section we present the multivariate autoregressive model (MAR) that was used to generate the simulated datasets. Next, a description of the Gaussian Mixture Expectation Propagation (GMEP) method is provided.

### Multivariate autoregressive model

Let **y**_*t*_ denote a *d*_*y*_ × 1 vector, representing the state of *d*_*y*_ time series measured at time *t*. A MAR model of order *p*, computes **y**_*t*_ as the linear combination of its *p* previous time points:

$$\begin{array}{c} y_{t}=\sum_{i=1}^{p}A_{i}^{\text{T}}y_{t-i}+e_{t}\;, \end{array}$$ (1)

where $e_{t}∼N\left(0,\text{diag}\left(\sigma_{1}^{2},...,\sigma_{d_{y}}^{2}\right)\right)$ is the so-called innovation process, its increments are temporally independent and each time instant is a realization from a *d*_*y*_-dimensional Gaussian distribution with zero mean and diagonal covariance matrix. The $A_{i}\in R^{d_{y}\times d_{y}}$ with *i* = 1,2,…,*p* are the coefficient matrices that model the influence of the signal values at time *t*−*i* on the current signal values at time *t*. Thus each **A**_*i*_ is involved in the data generating process associated with time lag *i*.

The so-called standard form of the model can be easily derived by constructing the (*d*_*y*_ *p*) × 1 vector $x_{t}={\left[y_{t-1}^{\text{T}},y_{t-2}^{\text{T}}\ldots y_{t-p}^{\text{T}}\right]}^{\text{T}}$. **x**_*t*_ contains the past dynamics of each time series needed to compute the current amplitude **y**_*t*_. All the **A**_*i*_ coefficient matrices of each time lag are vertically stacked in a unique (*d*_*y*_ *p*) × *d*_*y*_ matrix **W** = [**A**_1_;…;**A**_*p*_]. Thus, each **y**_*t*_ is equal to

$$\begin{array}{c} y_{t}=W^{\text{T}}x_{t}+e_{t}\;, \end{array}$$ (2)

which shows that the model can be identified by solving a multivariate linear regression problem.

### Gaussian scale Mixture Expectation Propagation method

We present a novel expectation propagation approach for sparse hierarchical generalized linear models and use it as a linear regression method for MAR model identification. Our approach was originally implemented in a more general way that allows the definition of various observation models and coefficient priors. Here, a summary of the method is presented in a context suitable for MAR modeling with a Gaussian observation model and a Gaussian scale mixture distribution for the group-sparsity prior. We will refer to it as GMEP. A detailed description of the model in its general form is given in the Supplementary Material.

As shown in Eq (2), for MAR modeling purposes it suffices to consider a linear regression problem with multiple output variables, where the probability density of each observed *d*_*y*_ × 1 output vector **y**_*i*_ depends on the *d*_*x*_ × 1 input vector **x**_*i*_ through a linear transformation **W**^T^ **x**_*i*_, and **W** is a *d*_*x*_ × *d*_*y*_ matrix of unknown coefficients. We assume that the observation noise is Gaussian and independent over different output variables as well as observations. Therefore, given *n* input-output pairs, denoted by $D={\left\{x_{i},y_{i}\right\}}_{i=1}^{n}$, the observation model can be written as

$$\begin{array}{ll} p(Y|XW,\theta ) & =\prod_{i=1}^{n}p(y_{i}|W^{\text{T}}x_{i},\theta )\\ & =\prod_{i=1}^{n}\prod_{k=1}^{d_{y}}N\left(y_{i,k}|w_{k}^{\text{T}}x_{i},\underset{=\sigma_{k}^{2}}{\underset{︸}{{\text{exp}}^{{}^{\circ}}\left(V_{j\left(i,k\right)}^{\text{T}}\theta \right)}}\right), \end{array}$$ (3)

where **Y** = [**y**_1_,…,**y**_*n*_]^T^ is a *n* × *d*_*y*_ output variable matrix, **X** = [**x**_1_,…,**x**_*n*_]^T^ is a *n* × *d*_*x*_ input variable (or design matrix) matrix, and **W** = [**w**_1_,…,**w**_*d*_*y*__] is a *d*_*x*_ × *d*_*y*_ coefficient matrix. The notation exp°(⋅) refers to element-wise exponential. In the case of a MAR model, index *i* enumerates all observed time instants up to *n*, and *d*_*y*_ corresponds to the number of interacting signals. We assume that each of the *nd*_*y*_ likelihood terms depends on the hyperparameters *θ* via a linear transformation by a known *d*_*θ*_ × 1 vector **V**_*j*(*i*, *k*)_ where *j*(*i*, *k*) = (*i*−1)*d*_*y*_ + *k*, and that the noise level for each output is encoded as $\sigma_{k}^{2}=\text{exp}{}^{\circ}\left(V_{j\left(i,k\right)}^{\text{T}}\theta \right)$. Here we simply assume that the noise level can differ between signals but that the noise variance is constant over time points. This can be achieved by including one noise parameter for each output in *θ* and by making **V**_*j*(*i*,*k*)_ a binary vector that picks the desired component from it for each likelihood term.

The hierarchical prior distributions is of the form $p(W|\theta )\propto \prod_{j=n+1}^{n+m}p(U_{j}^{\text{T}}w|V_{j}^{\text{T}}\theta )$, where **w** = vec(**W**) is a *d*_*w*_ × 1 coefficient vector obtained by vertically concatenating the columns of **W**. The known transformation matrices **U**_*j*_ and **V**_*j*_ are assumed to yield low-dimensional scalar random variables suitable for efficient inference using EP. For MAR identification we adopt a structured Gaussian scale-mixture prior of the form

$$\begin{array}{cc} p(W|\theta ) & =\prod_{k=1}^{d_{y}}\prod_{l=1}^{d_{x}}N\left(w_{l,k}|0,\text{exp}{}^{\circ}\left(V_{j\left(l,k\right)}^{\text{T}}\theta \right)\right), \end{array}$$ (4)

where *j*(*l*, *k*) = *n*+(*k*−1)*d*_*x*_+*l* and the prior variance of coefficient *w*_*l*,*k*_ is controlled by $\text{exp}{}^{\circ}\left(V_{j\left(l,k\right)}^{\text{T}}\theta \right)$. In GMEP this is obtained by setting **U**_*j*_ to be unit vectors that pick only one coefficient at a time and *j* to be binary indicator vectors that cluster the coefficients into a certain number *n*_*g*_ of predefined groups. Each of the groups is assigned an unknown variance hyperparameter exp°(*θ*_*g*(*j*)_) that is picked up by the inner product $\theta_{g\left(j\right)}=V_{j}^{\text{T}}\theta$ for each coefficient.

We assign a fixed multivariate Gaussian prior density to the hyperparameters ***θ***:

$$\begin{array}{c} p(\theta )=N(\mu_{\theta ,0},\Sigma_{\theta ,0}), \end{array}$$ (5)

where ***μ***_*θ*,0_ is the hyperprior mean vector and Σ_*θ*,0_ the hyperprior covariance matrix. By adjusting ***μ***_*θ*,0_ and Σ_*θ*,0_ we can form coefficient priors with different sparsity-promoting properties. For example, if we set **V**_*j*(*l*,*k*)_ to unit vectors that attach only one hyperparameter to each coefficient and assume Σ_*θ*,0_ to be diagonal, we can create sparser solutions by increasing the diagonal entries of Σ_*θ*,0_ and decreasing the prior means ***μ***_*θ*,0_. An uninformative signal-specific noise prior can be obtained by making the corresponding elements of ***μ***_*θ*,0_ sufficiently small and including an “independent” diagonal block in Σ_*θ*,0_ with sufficiently large diagonal values. This corresponds to setting independent log-normal priors to the noise variances $\sigma_{k}^{2}$.

**Fig 1:**
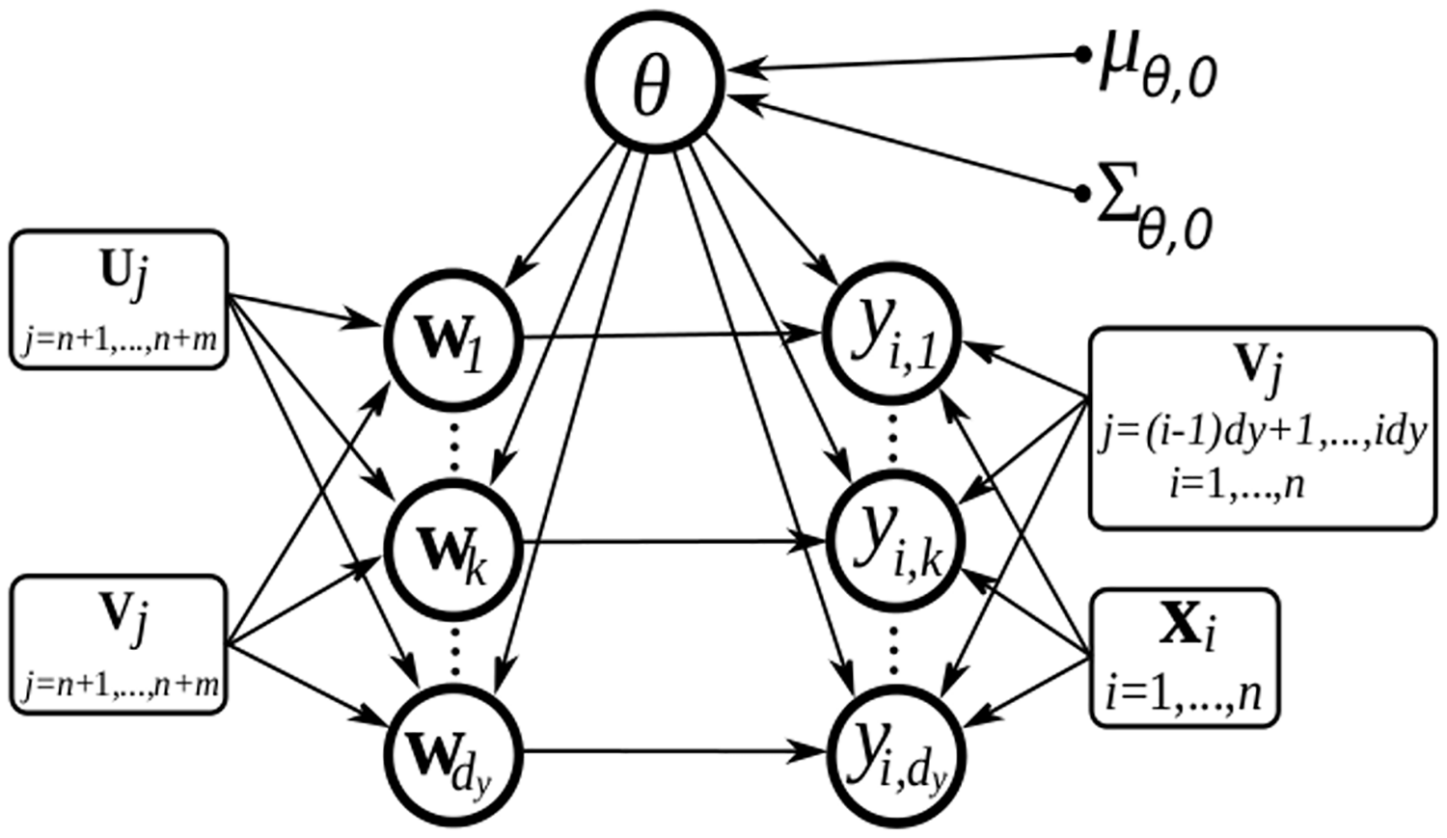
Graphical model of GMEP in which dependences between variables are shown by using circles for random variables, rectangles for known variables and dots for fixed hyperparameters.

Fig 1 shows the graphical model representation of GMEP. Random variables are denoted with circles, while known variables are denoted with rectangles. The fixed hyperpameters ***μ***_*θ*,0_ and Σ_*θ*,0_ are denoted with dots.

This general model definition enables the implementation of various different linear models via the choice of the transformations **V**_1_,…,**V**_*n*_ for the likelihood terms and **V**_*n* + 1_,…,**V**_*n* + *d*_*y*_*d*_*x*__ for the prior terms. In the following we present the three structured coefficient priors that will be used in the experiments. They are described in a formal and mathematical way by considering the notation adopted until now, their actual interpretation and meaning will be discussed later in Subsection “Employed structured coefficient priors”.

#### Uniform Gaussian prior

A uniform Gaussian prior with unknown scalar prior variance for each output (similar to ridge regression) can be obtained by choosing **U**_*j*_ = **e**_*j*_ (*d*_*w*_ × 1) and **V**_*j*_ = **e**_*k*_ (*d*_*y*_ × 1) for *j* = (*k* − 1)*d*_*x*_ + *n* + 1,…,(*k* − 1)*d*_*x*_ + *n* + *d*_*x*_ and *k* = 1,..,*d*_*y*_. This leads to *n*_*g*_ = *d*_*y*_ different inference problems, if the coefficients related to different outputs are not coupled through the observation model;

#### Automatic relevance determination

An automatic relevance determination (ARD) prior can be formed by assigning individual scale hyperparameters to each coefficients. Thus, we set **U**_*j*_ = **V**_*j*_ = **e**_*j*_ (*d*_*w*_ × 1) with *j* = *n* + 1,…,*n* + *d*_*w*_. This construction assumes individual scale parameters for each coefficient *n*_*g*_ = *d*_*w*_ and no information sharing between the outputs, which results in independent regression problems for each output. This prior is very flexible because each of the *d*_*w*_ = *d*_*y*_ *d*_*y*_ *p* coefficients can be regularized out of the model independently, but the resulting inference problem is also more challenging in terms of avoiding overfitting.

#### Group sparsity prior

Group sparsity priors can be constructed by defining possibly overlapping groups as **U**_*j*_ = **e**_*j*_ (*d*_*w*_ × 1) and **V**_*j*_ = [1, 0, 1, 0, 0,…,0]^T^ (*n*_*g*_ × 1). Groups could be defined either so that they combine coefficients from different output units into same groups or completely separately for each output. In particular, in our experiments we will use a group sparsity prior defined by choosing **U**_*j*_ = **e**_*j*_ (*d*_*w*_ × 1) and **V**_*j*_ = **e**_(*r* − 1)*d*_*y*_ + *l*_ (*d*_*y*_ *d*_*y*_ × 1) with *j* = (*k* − 1)*d*_*y*_ + (*r* − 1)*d*_*x*_ + *l* + *n* for *l* = 1,…,*d*_*y*_, *r* = 1,..,*d*_*y*_ and *k* = 1,…,*p*. From now on, we will refer to this group sparsity prior as lag-independent sparsity since it assumes that the coefficient sparsity structure is independent from the time lag and also not shared between the outputs. Compared to the ARD prior, the lag-independent sparsity is less flexible because it combines information over different lags. However, it still provides *d*_*y*_ × *d*_*y*_ free prior parameters that can explain the causality structure between the *d*_*y*_ interacting signals in our MAR model.

### Approximate inference

The GMEP posterior density *p*(**w**, *θ*|**Y**, **X**), is formed by multiplying together Eqs (3), (4) and (5), and normalizing the result with the marginal likelihood *Z* = *p*(**Y**|**X**) (or the model evidence):

$$\begin{array}{c} p\left(w,\theta |Y,X\right)=Z^{-1}p\left(y|W,\theta ,X\right)p\left(W,|\theta \right)p\left(\theta \right). \end{array}$$ (6)

Because the posterior density is not analytically tractable, a deterministic approximation to it is computed using the EP algorithm [34]. To simplify the notation for the remainder of this section, we define

$$g_{i}\left(w,\theta \right)=\{\begin{array}{ll} N(y_{i,k}|w_{k}^{\text{T}}x_{i},\text{exp}{}^{\circ}\left(V_{j_{\left(i,k\right)}}^{\text{T}}\theta \right)), & \text{if }i=1,\ldots ,nd_{y}\\ N(w_{l,k}|0,\text{exp}{}^{\circ}\left(V_{j_{\left(i,k\right)}}^{\text{T}}\theta \right)), & \text{if }i=nd_{y}+1,\ldots ,nd_{y}+d_{y}d_{x}, \end{array}$$ (7)

where *i* is a now a generic index that enumerates all the intractable likelihood and prior terms. A joint Gaussian posterior approximation denoted by *q*(**w**, *θ*), is formed by replacing the non-Gaussian likelihood and prior terms with joint Gaussian functions of **w** and *θ*:

$$\begin{array}{c} p(w,\theta |Y,X)=Z^{-1}\prod_{i=1}^{n_{q}}g_{i}\left(w,\theta \right)p\left(\theta \right)\approx q\left(w,\theta \right)=Z_{\text{EP}}^{-1}\prod_{i=1}^{n_{q}}{\tilde{g}}_{i}\left(w,\theta \right)p\left(\theta \right) \end{array}$$ (8)

where *n*_*q*_ = *nd*_*y*_ + *d*_*y*_ *d*_*x*_, and ${\tilde{g}}_{i}\left(w,\theta \right)$ are scaled local Gaussian model term approximations each with a different scale, location and precision parameter (see the appendix for details). No local approximation is needed for the prior *p*(*θ*), because it is already Gaussian. Also, if *θ* was known, no EP approximation would be needed since the model terms *g*_*i*_(**w**, *θ*) are already Gaussian with respect to **w**.

The EP algorithm starts by initializing the approximate factors ${\tilde{g}}_{i}\left(w,\theta \right)$ to some sensible values. In practice, the likelihood terms can be initialized to one, i.e., the location and precision parameters can be set to zero so that they effectively disappear from the approximation *q*(**w**, *θ*). The prior term approximations can be initialized to a regularizing ridge-like prior, where the location parameters are zero and the precision terms are set to some small positive values.

The standard EP algorithm proceeds by updating each term approximation in turn. At each update, first, one of the approximate terms is removed from the approximation to form a cavity distribution

$$\begin{array}{c} q_{-i}\left(w,\theta \right)\propto {\tilde{g}}_{i}{\left(w,\theta \right)}^{-1}q\left(w,\theta \right), \end{array}$$ (9)

which for the likelihood terms can be regarded as an approximation to the leave-one-out posterior density. Next the removed term approximation is replaced with the actual model term to give a tilted distribution, which can be regarded as a more refined approximation to the posterior:

$$\begin{array}{c} {\hat{p}}_{i}\left(w,\theta \right)={\hat{Z}}_{i}^{-1}g_{i}\left(w,\theta \right)q_{-i}\left(w,\theta \right), \end{array}$$ (10)

where the normalization term is given by

$$\begin{array}{c} {\hat{Z}}_{i}=\int g_{i}\left(w,\theta \right)q_{-i}\left(w,\theta \right)dwd\theta . \end{array}$$ (11)

For the likelihood terms, the normalization variables ${\hat{Z}}_{i}$ can be regarded as an approximation to the leave-one-out predictive density for the corresponding data point. Then the parameters of the left-out approximate term are updated so that the KL divergence from the tilted distribution to the true approximate posterior is minimized:

$$\begin{array}{c} {\tilde{g}}_{i}{\left(w,\theta \right)}^{\text{new}}=\underset{{\tilde{g}}_{i}}{arg min}\text{KL}({\hat{p}}_{i}\left(w,\theta \right)||{\tilde{g}}_{i}\left(w,\theta \right)q_{-i}\left(w,\theta \right)). \end{array}$$ (12)

In the case of a Gaussian approximation this corresponds to matching the mean and the covariance of the approximation with the corresponding moments of the tilted distribution. After a chosen subset of the model term approximations have been updated according to Eq (12), also the posterior approximation *q*(**w**, *θ*) is recomputed.

These steps are repeated at some order for all model terms until convergence. In practice we update all the likelihood terms in one batch keeping the prior term approximations fixed, and vice versa. Finally, after convergence, posterior summaries of the unknown model parameters and predictions are computed using the Gaussian approximation *q*(**w**, *θ*).

## Materials

The first two parts of this section describe the simulated and empirical datasets that were used in the experiments. Whereas the last part is about the structured coefficient priors adopted in GMEP.

### Simulated MAR datasets

The synthetic datasets were generated by an MAR model, and our goal is to study how good is the identification of GMEP. In order to explore the model performance in different regimes, multiple ensembles of time series were generated under different conditions. In our simulations the free parameters that identify a dataset are the dimensionality *d*_*y*_ and the connection density *c*. Here, *d*_*y*_ refers to the number of time series contained in each trial of the dataset and *c* refers to the fraction of non-zero off-diagonal connections (i.e. causal interactions). This choice to characterise each dataset through the pair (*d*_*y*_, *c*) is motivated by the fact that it heavily influences the ability to accurately estimate causal interactions.

In our simulations *d*_*y*_ ∈ {3, 7, 11} and *c* ∈ {0.1, 0.5, 0.9}. Each dataset, indexed by (*d*_*y*_, *c*), consists of 100 trials (repetitions). Each trial **Y** = [**y**_1_,…,**y**_*n*_]^T^ is a *n* × *d*_*y*_-dimensional matrix, where the length of each to the *d*_*y*_ time series is set to *n* = 1500 time points. **Y** is generated by an MAR model of the predefined order *p* = 10 and with a predefined causal configuration matrix **A**. **A** is a binary matrix, it contains the causal structure that determines the interactions between time series. Specifically, **A**(*r*, *s*) = 1 means that signal *r* causes signal *s*. In each time lag, the related **A**_*i*_ matrix is generated by multiplying the non-zero elements of **A** with uniformly distributed random numbers. Such uniform distribution is centered on zero and its width is defined in order to guarantee the stability of the model. Thus, the distribution is shaped according to *d*_*y*_ and *c*. In details, after having centered the distribution on zero, it is scaled by a factor *k* that is initialized to 2.2 and then increased with a step of 0.05 if among 1000 different models none of them is stable. For each pair (*d*_*y*_, *c*), Table 1 reports the scaling factor *k* that allowed at least one stable model among 1000.

**Table 1:** Scaling factor *k* used to generate stable trials given their dimensionality *d*_*y*_ and connection density *c*.

		*c*		
		0.1	0.5	0.9
*d*_*y*_	3	2.2	2.2	2.2
	7	2.2	2.3	3.0
	11	2.2	3.0	3.8

Each trial has its own configuration matrix **A** while the connection density *c* is shared between trials in the same dataset. Not all the *n* time points are used in the analyses since we decided to keep the same proportion of elements in the design matrix and unknowns (coefficients) in order to have more comparable results across datasets. Thus the number of actual time points involved in the experiments depends on *d*_*y*_. In Table 2, we report for each *d*_*y*_ the related *n* and the resulting shape of **Y**, **X** and **W**.

**Table 2:** For each *d*_*y*_ the number of time points *n* is specified and the resulting shape of matrices Y, X and W.

*d*_*y*_	*n*	Y: [*n* × *d*_*y*_]	X: [*n* × (*d*_*y*_ *p*)]	W: [(*d*_*y*_ *p*) × *d*_*y*_]
3	189	189×3	189×30	30×3
7	441	441×7	441×70	70×7
11	693	693×11	693×110	110×11

In the last experiment conducted on the simulated data we introduced a third free parameter: the level of noise *γ*, *γ* ∈ {0.2, 0.4, 0.6, 0.8}. Each trial is computed as **Y** + *γ***Y_noise_**, where **Y_noise_** has the same shape of **Y** and it is the output of an univariate AR process.

### Empirical fMRI dataset

The empirical data we used belong to the *Gallant Lab Natural Movie 4T fMRI Dataset* [37, 38] and were acquired on a 4T Varian INOVA scanner. The scanning was done using T2*-weighted gradient echo EPI: TR = 1 s, TE = 28 ms, Flip angle = 56 degrees, voxel size = 2.0 × 2.0 × 2.5 mm^3^, and FOV = 128 × 128 mm^2^. A total of 18 coronal slices were acquired and they cover the posterior portion of occipital cortex, starting at the occipital pole. A parcellation of the measured voxels into 26 regions of interest was provided by the authors. Subjects were presented with natural movies during a training and a test session. See [38] for further details about the experimental protocol.

The time series we used in our analysis were extracted from the training dataset of one of the three acquired subjects by averaging signals corresponding to the same region of interest. This gave for each subject 26 time series; one for each ROI. Each time series had a length of 7200 s, since 12 separate 10-minute blocks of movies were presented for the training dataset. For our analyses, we considered the concatenated block and ignored modelling errors at the boundaries between blocks.

### Employed structured coefficient priors

We have previously explained the structured priors in analytical terms, here we recall them giving an interpretation of their analytical definition from the point of view of the sparsity structure that they assume.

Firstly, a uniform Gaussian prior was defined for each output. Such configuration is strictly related to ridge regression because the coefficients associated at each output are supposed to belong to the same group that means they are modelled as drawn from the same distribution. This implies that sparsity is shared across all coefficients in the same column of **W** since only the hyperparameters that define such distribution tune the level of sparsity. In other words, we can see this as the GMEP implementation of ridge.

The second trivial configuration that was taken into account, considers one group for each coefficient. It represents the opposite situation with respect to the previous prior, thus now each coefficient has its own distribution to which it belongs to. This approach is known as automatic relevance determination (ARD) because the hyperparameters of each distribution determine the sparsity i.e. the relevance, of the related coefficient.

The third case in our comparison has a definition of groups that reproduces the true sparsity structure of the coefficients in **W**. Referring to Eq 1 and to the description of how each **A**_*i*_ was computed from **A**, we can see that the same sparsity structure is shared across time lags, i.e. the amount and position of the zero connections are the same across **A**_**i**_. This assumption can be rephrased as: the causal configuration is time independent, i.e. there is no dynamic in the causal interactions. Therefore, we call that prior group the lag-independent prior.

## Experiments

This section describes the experiments that were run to analyse GMEP and to study its application both on simulated and empirical data.

### Simulated MAR datasets

We start with the experiments that were run on the synthetic data. As described below, these experiments have three unique purposes.

The first purpose is to compare GMEP and other standard linear regression approaches. In particular, we refer to Ordinary Least Squares (OLS), Levinson-Wiggs-Robinson equations (LWR) and Ridge Regression (RR). They are all standard methods widely used for linear regression. In particular, OLS and LWR are both used in practice to fit the MAR parameters in MVGC. Moreover, both are point estimator methods and asymptotically equivalent to the maximum likelihood estimate. The last technique, RR, is included since it contains a regularization term in order to prevent overfitting.

Note that LWR derives from a multivariate extension to Durbin recursion and it has the advantage to provide also an estimate of the residual covariance matrix $\hat{\Sigma }$. For further details refer to [15, 39]. RR can be simply expressed by adding the *l*_2_-norm of the coefficient matrix **W** in the objective function of OLS [40]. In this way, the magnitude of the coefficient is included in the minimization process and it is forced to be small according to a weight parameter that controls the amount of shrinkage. The comparison of GMEP, OLS, LWR and RR is done by running them on each synthetic dataset and focusing on their capability to estimate the coefficient matrix. The model order is set equal to its true value, i.e. *p* = 10, and the performance of each approach is evaluated through the normalized root mean square error (NRMSE) computed between the true and the mean of posterior distribution of the estimated coefficients. The normalization is done according to the maximum amplitude (the difference between the maximum and minimum) of the true coefficients. Hence, NRMSE is not necessarily bounded in [0, 1]. Regarding the model order, beyond the commonly used information criteria such as the Akaike information criterion or the Schwarz criterion, it can be estimated within the Bayesian GMEP framework using either the marginal likelihood estimate (model evidence) or the leave-one-out predictive density estimate. We tested this model order selection on the simulated dataset and it will be applied on the fMRI analysis.

The second purpose of the experiments on the simulated data is to focus exclusively on GMEP and analyse the impact of the structured priors. In particular, the aim is to show that a more detailed modelling of the group sparsity prior, through the inclusion of information related to the structure of the data, improves the results. Thus, we are interested in proving that there are situations in which an accurate definition of the structured prior leads to a better inference. This improvement is observable not only through a comparison with the true coefficients but also by evaluating the reconstructed time series. To understand how the structured priors affect the final results, a comparison across three different priors is conducted.

The predictive performances of the different priors are evaluated by computing the mean log predictive densities (MLPD):

$$\begin{array}{cc} & \frac{1}{n_{t}d_{y}}\sum_{i=1}^{n_{t}}\sum_{j=1}^{d_{y}}\log\int p\left(y_{i,j}^{*}|w_{j}^{\text{T}}x_{i}^{*},\text{exp}{}^{\circ}\left(v_{i,j}^{\text{T}}\theta \right)\right)p\left(w,\theta \right|D)dwd\theta \\ & \approx \sum_{i=1}^{n_{t}}\sum_{j=1}^{d_{y}}\log\int p\left(y_{i,j}^{*}|w_{j}^{\text{T}}x_{i}^{*},\text{exp}{}^{\circ}\left(v_{i,j}^{\text{T}}\theta \right)\right)q\left(w_{j}\right)q\left(\theta \right)dw_{j}d\theta , \end{array}$$ (13)

where $y_{i}^{*}={\left[y_{i,1}^{*},...,y_{i,d_{y}}^{*}\right]}^{\text{T}}$ is a known test observation at test input $x_{i}^{*}$, and *q*(**w**, ***θ***) = ∏_*j*_ *q*(**w**_*j*_)*q*(***θ***) is given by the EP approximation. With a Gaussian observation model the required integrals can be computed using one dimensional numerical quadratures. Higher MLPD values correspond to higher approximate predictive density values for test data points on average indicating thus better predictive performance.

The normalization coefficients ${\hat{Z}}_{i,j}$ of the tilted distributions Eq (10) are obtained as by-products of the EP algorithm. Since the cavity distributions *q*_−*i*_(**w**, ***θ***) can be regarded as an approximation to the posterior when observation *y*_*i*,*j*_ is left out from the training set, we can use the normalisation terms ${\hat{Z}}_{i,j}$ to form an approximation to the mean leave-one-out predictive densities:

$$\begin{array}{c} {\text{MLPD}}_{\text{EP}}=\frac{1}{nd_{y}}\sum_{i=1}^{n}\sum_{j=1}^{d_{y}}\log{\hat{Z}}_{i,j}. \end{array}$$ (14)

In the experiments we use MLPD_EP_ as an estimate of the future predictive performance of the model and validate it with respect to the actual MLPD score using simulated experiments.

For a known coefficient vector w*, a similar measure that we call MLPD_**w**_ can be computed as

$$\begin{array}{c} {\text{MLPD}}_{w}=\log\int p\left(w^{*},\theta \right|D)\theta \approx \sum_{j=1}^{d_{y}}\log q\left(w_{j}^{*}\right), \end{array}$$ (15)

which measures how well the posterior approximation matches with the true coefficients. A higher MLPD_**w**_ value indicates a better agreement with the EP posterior approximation *q*(**w**) and the true coefficients w*.

The third purpose of the experiments conducted on the simulated data is to obtain an estimate of the binary causal configuration matrix from the results of GMEP. Such analysis requires the choice of a specific structured prior and a way to obtain the binary causal configuration matrix from the results of the inference process. The proper structured prior is chosen according to the outcome of the previous experiment by selecting the one with the best results, as we will see later it is the lag-independent prior. And the binary causal configuration matrix is computed by considering that an estimate of the variance distribution of each group is provided by GMEP. In detail, due to the choice of the lag-independent prior each group contains all the coefficients that link the same pair of time series at different time lags. Thus, there is one group for each cell of the binary configuration matrix. Moreover, the coefficients in each group are supposed to be normally distributed with zero mean and variance that is modelled as a log-normal distribution. After the estimation process, the posterior mean and standard deviation of such distribution are used to reconstruct the causal configuration matrix of each trial by their normalization and comparison. The causal configuration matrices predicted by MVGC and the ones predicted by GMEP are evaluated with the related ground truth. Such comparison is extended also to the datasets with the noise component, thus to all the (*d*_*y*_, *c*, *γ*) datasets. From the estimated causal configuration matrix of each trial, the true positive rate and true negative rate are computed and averaged across trials with the same level of noise. This procedure was repeated both for MVGC and GMEP, in order to compare them in term of their balanced accuracy (BA). We chose the balanced accuracy, as evaluation measure since it overcomes the problem of unbalanced dataset [41]. BA is meant as the mean of the true positive rate and the true negative rate across all the trials in each (*d*_*y*_, *c*, *γ*) dataset.

### Empirical fMRI dataset

The second part of the experiments focuses on the empirical data. We are aware of the existing debate about using time lag-based method with fMRI data. Indeed a number of studies state that the BOLD response is not compatible with the assumptions of precedence and predictability that are at the root of Granger causality [42, 43], while others prove the robustness of Granger causality to variations of the hemodynamic response function and identifies the noise level and the amount of downsampling as possible issues in causal prediction [44]. Here, we do not enter this debate but we aim to use the Bayesian model as a way to test hypotheses about the sparsity structure. In this way, if prior knowledge is available on the structure of the data, then it is possible to test it and to compare the results with respect to a baseline case such as the ARD or the uniform Gaussian priors.

When working with empirical data, the main difference with respect to the analysis conducted on the simulated one, is that we do not have the ground truth on the sparsity structure. This lack of information can be replaced by prior knowledge on the data. For example, it is reasonable to assume a difference in the magnitude of the coefficients that connect areas in the same hemisphere respect to the ones that connect areas across hemispheres. Such an assumption can be encoded in a specific group sparsity prior and a comparison with other structured priors can reveal which is the closest to the ground truth. We defined an experiment in which the length of each time series is gradually reduced in order to compare the performances of GMEP under both different structured priors and number of training time points. In detail, the experiment that we have carried out on the empirical dataset, is the following: first, part of the dataset is used to identify the best model order with a grid search approach, i.e. for each *p* in [2: 2: 14] a MAR model was identified and we selected the order which provided the best identification. Next, we apply GMEP using the three structured priors that we adopted on the simulated data. Moreover, in the definition of the structured prior we also consider the anatomical position associated with each time series. That is, we enrich the three initial priors by adding four new groups in which the coefficients are clustered according to the hemispheres that they link with. The results of these two scenarios, i.e. the three structured priors and their enrichment with anatomical information, are compared with the prior that only models the hemisphere structure. This allow us to identify the most plausible group sparsity prior among the tested ones. This prior is used in the final analysis, where the aim is to compute the causal configuration matrix by using GMEP. The approach used to obtain a binary matrix is the same as the one used for the simulated data based on the comparison between the posterior mean and posterior standard deviation of the group variances.

## Results

Results are divided according to the dataset from which they were obtained, thus the first part of this section is devoted to the findings from the simulated MAR datasets and the latter to the empirical fMRI dataset.

### Simulated MAR datasets

**Fig 2:**
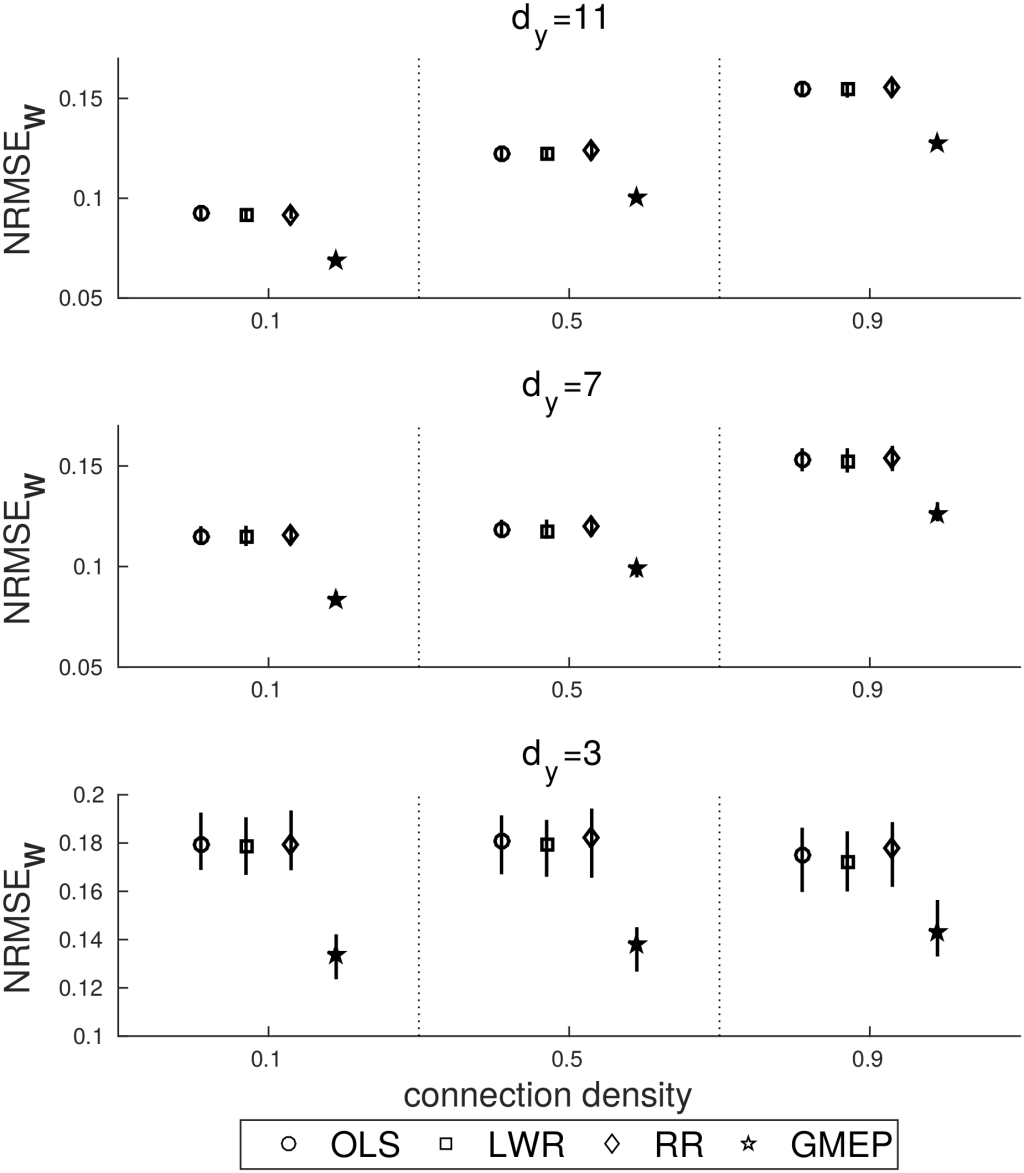
NRMSE related to the coefficient estimations, each inference method is identified by a specific marker and its result is reported in terms of median, 25-th and 75-th percentiles.

Fig 2 shows the results of the NRMSE computed between estimated and true coefficients by OLS, LWR, RR and GMEP. These four methods were applied at each simulated dataset. The figure reports the median and the 25-th and 75-th percentiles computed on the 100 trials of each dataset. In the case of RR the strength of the regularization term *λ* was selected through a grid search approach applied on a subset of time points, i.e. for each *λ* in [0: 0.1: 100] and the choice was based on the RMSE. For GMEP the uniform Gaussian coefficient prior was adopted. The results show that, while the prediction errors of OLS, LWR and RR do not show large differences, the prediction error of GMEP is consistently better. As expected, we observe that NRMSE increases with the connection density. Moreover, the percentiles are very small thus the prediction error is stable across trials in each dataset and for each regression method.

**Fig 3:**
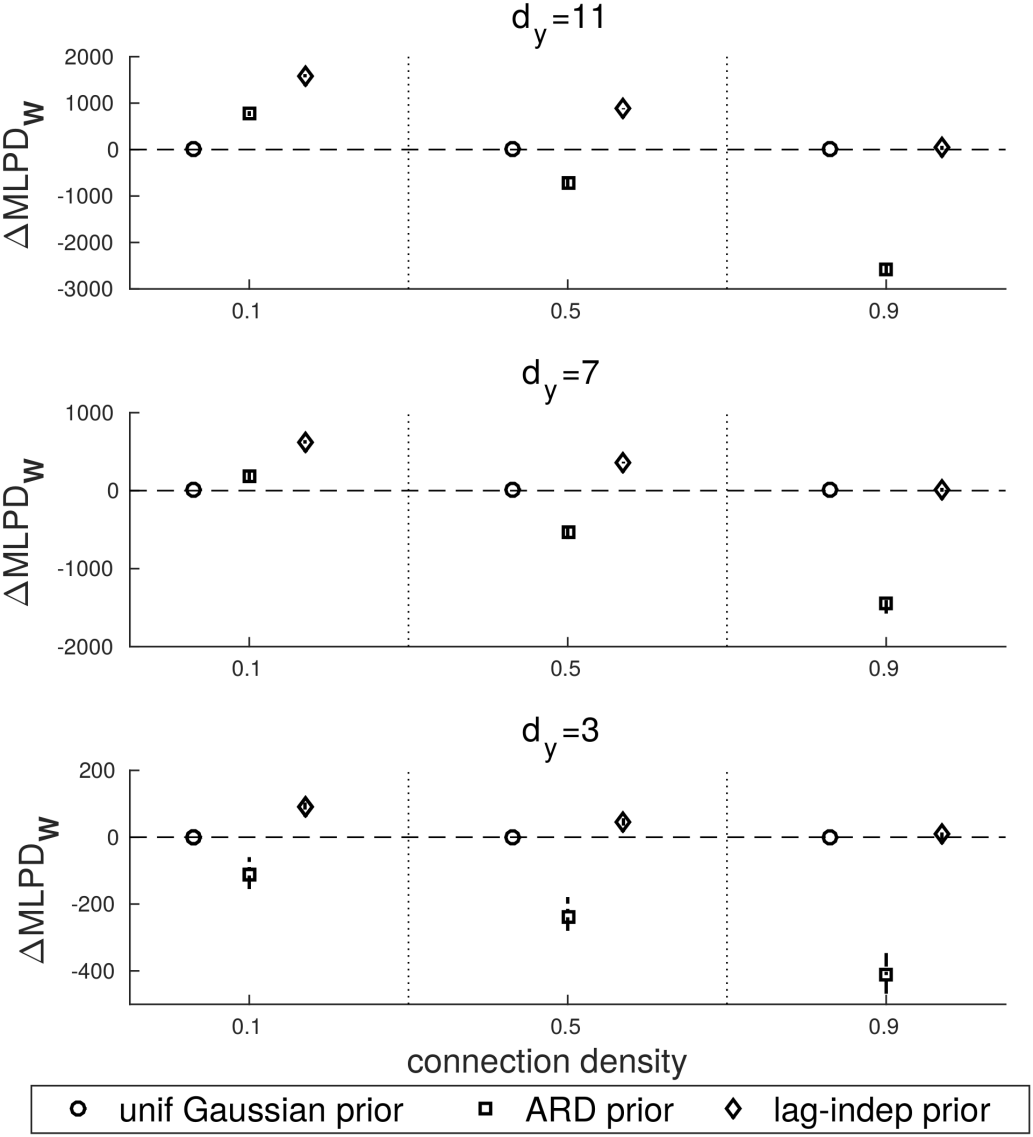
ΔMLPD computed with respect to the uniform Gaussian prior and evaluated on the coefficient estimates.

**Fig 4:**
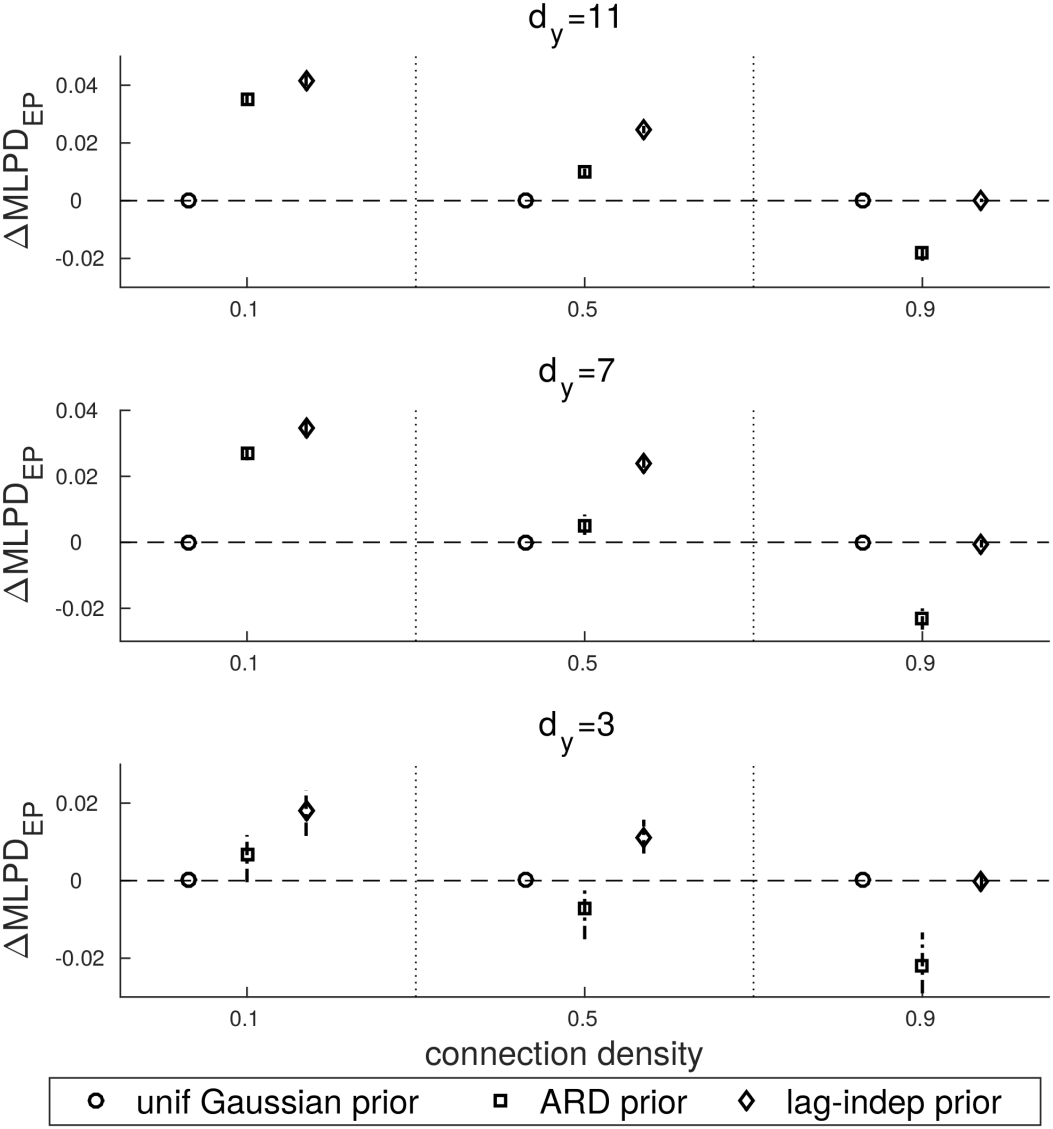
ΔMLPD computed with respect to the uniform Gaussian prior and evaluated on the EP iterations.

**Fig 5:**
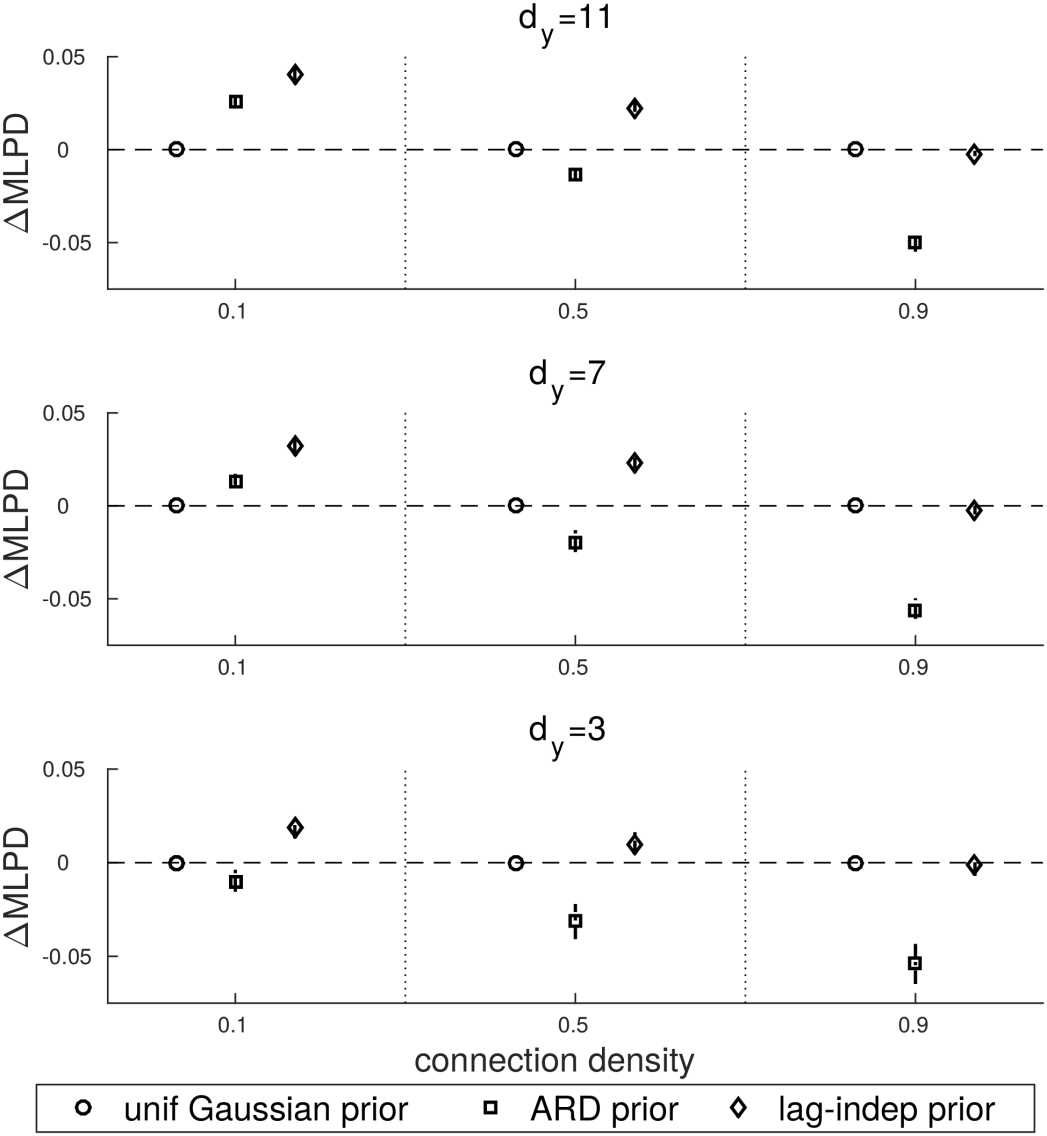
ΔMLPD computed with respect to the uniform Gaussian prior and evaluated on the test set.

Next, we analysed the performance of GMEP under different coefficient priors. The comparison across priors is done by using the uniform Gaussian prior as a baseline with which other priors are compared. The predictive performance is evaluated through the mean log predictive density (MLPD). In particular, we will consider the variation of MLPD with respect to the uniform Gaussian prior that we indicate as ΔMLPD. In Fig 3 the ΔMLPD_**W**_ computed on the coefficients is shown. Fig 4 contains the ΔMLPD_EP_ and Fig 5 the actual ΔMLPD computed on a separated test set.

Fig 3 shows that the ARD prior outperforms the uniform Gaussian prior only for connection density equals to 0.1 and dimensionality equals to 7 and 11. Its performance decreases as connection density is increased. In general, the lag-independent prior performs better than the other priors, particularly for low to medium connection densities. The lag-independent prior becomes comparable to the uniform Gaussian prior in the case of very dense configurations.

The same behaviour is reported in Figs 4 and 5. Both the ARD and the lag-independent priors get worse with the increase of the connection density with the difference that the lag-independent prior becomes comparable to the uniform Gaussian prior in the worst case. On the other hand the ARD prior drops faster and only in few cases it is better than the uniform prior. By comparing Figs 4 and 5 the generalization capability of the ARD and the lag-independent priors is highlighted. In fact we notice that in the ARD prior the MLPD drops faster than the MLPD_EP_ but in the case of the lag-independent prior MLPD and MLPD_EP_ behave similarly.

**Fig 6:**
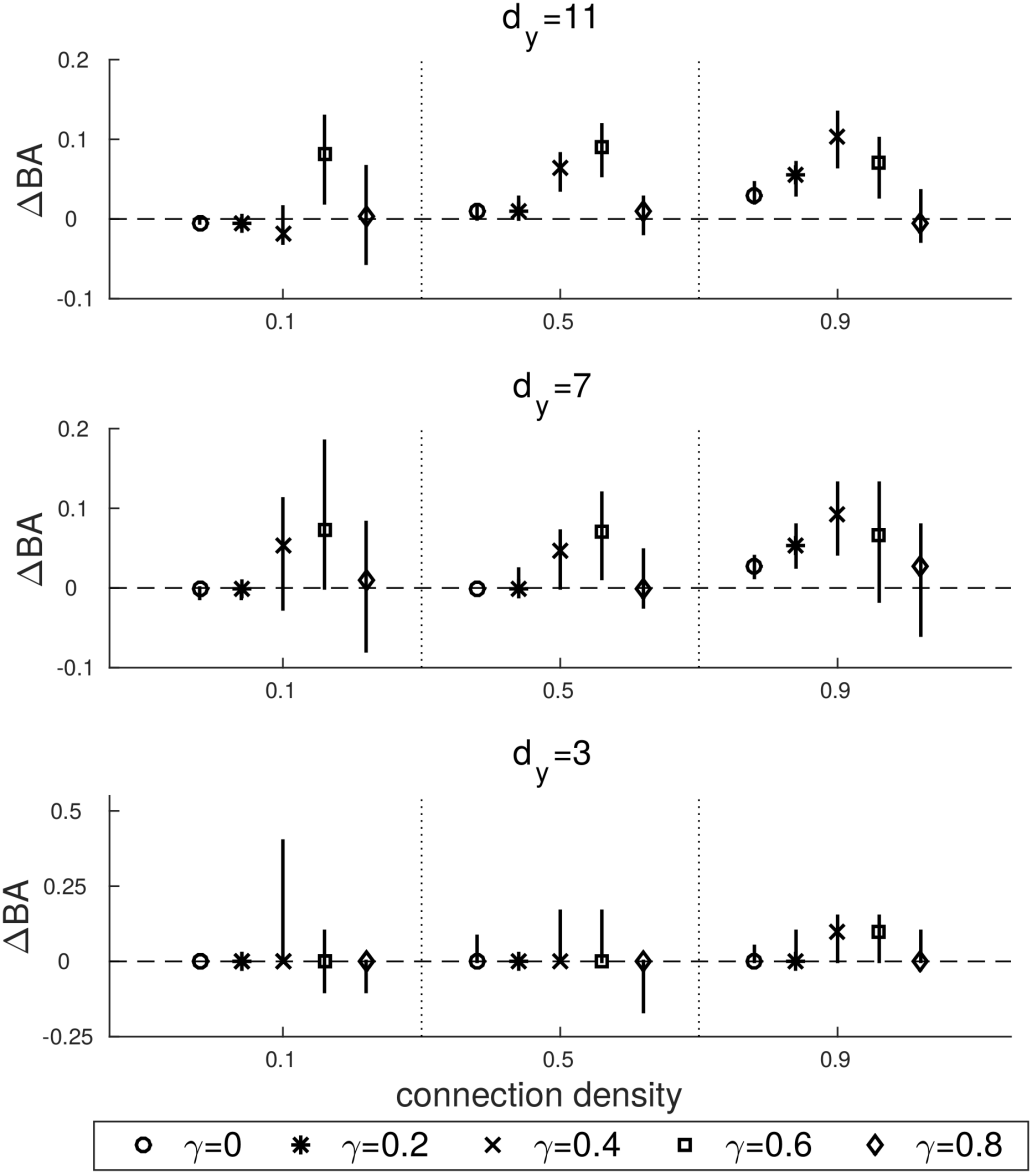
ΔBA computed on the causal configuration matrices estimated by GMEP and MVGC.

Fig 6 shows the difference between the balanced accuracy computed by applying GMEP and MVGC, under different levels of noise. We remember that the balance accuracy BA is defined as the mean of the true positive rate and the true negative rate and we will refer at the difference of BA between GMEP and MVGC as ΔBA. The noise level is quantified by the parameter *γ* and indicates the proportion between the actual signal and the univariate noise, i.e. *γ* = 0 means that the noise component is absent. As in the previous figures, the median and the 25-th and 75-th percentiles are reported. In this case the marker indicates the level of noise. The figure shows that there are no meaningful differences in the cases of *d*_*y*_ = 3, i.e. for low dimensionality. On the other hand, significant differences appear when the noise level increases and in particular when also the connection density increases. At increasing noise levels, the predictions of GMEP become more accurate than the predictions of MVGC. The gap of BA between the two approaches reaches the 10% in favour of GMEP for medium levels of noise (*γ* = 0.4 and *γ* = 0.6) and it drops to 0 when the data are dominated by the noise, i.e. *γ* = 0.8.

### Empirical fMRI dataset

**Fig 7:**
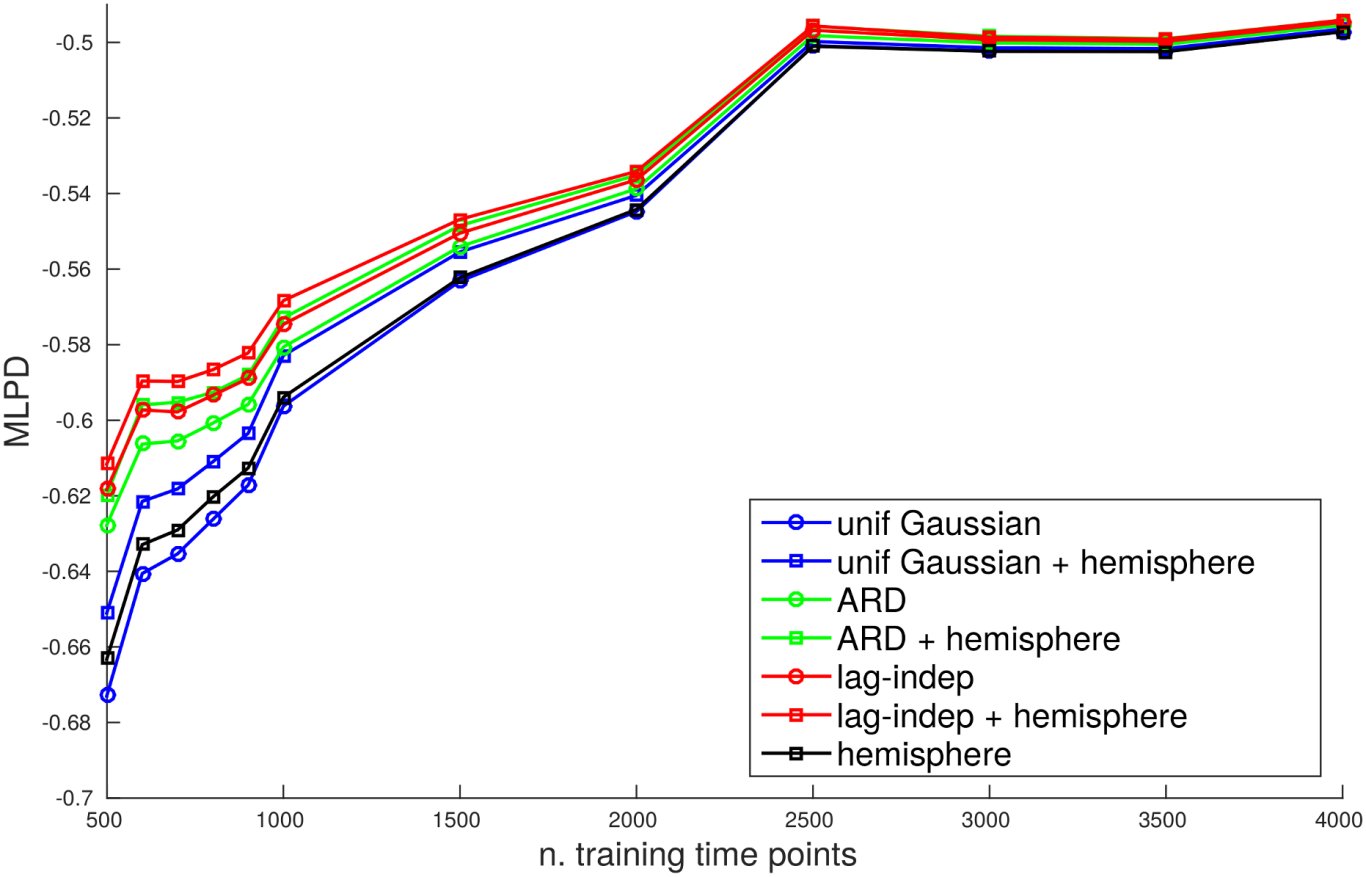
MLPD on the test set computed by multiple applications of GMEP under different structured priors and by varying the number of time points in the training set.

The experiments conducted on the empirical data are meant to test hypotheses about the sparsity structure of the causal interactions among the brain regions which the analysed time series correspond to. In Fig 7, we report the MLPD under different structured priors and number of training time points. We did not include the equivalent results for other performance measures since they show the same trend. In detail, the first 500 time points were firstly used to determine the order of the MAR model. This analysis showed a good compromise between performance and model complexity for *p* = 4. Using this model order, GMEP was applied in conjunction with the uniform Gaussian prior, the ARD prior and the lag-independent sparsity prior. Fig 7 reports with lines marked by circles the results of these priors using a different colour for each of them. The lines marked by squares show the effect of the inclusion of the partitioning based on the hemispheres. The black line reports the results with only the hemisphere groups in the sparsity structure prior. The results always show an improvement when the hemisphere groups are included in the structured prior. Moreover, consistent with the simulations, the lag-independent prior achieved the highest performance.

**Fig 8:**
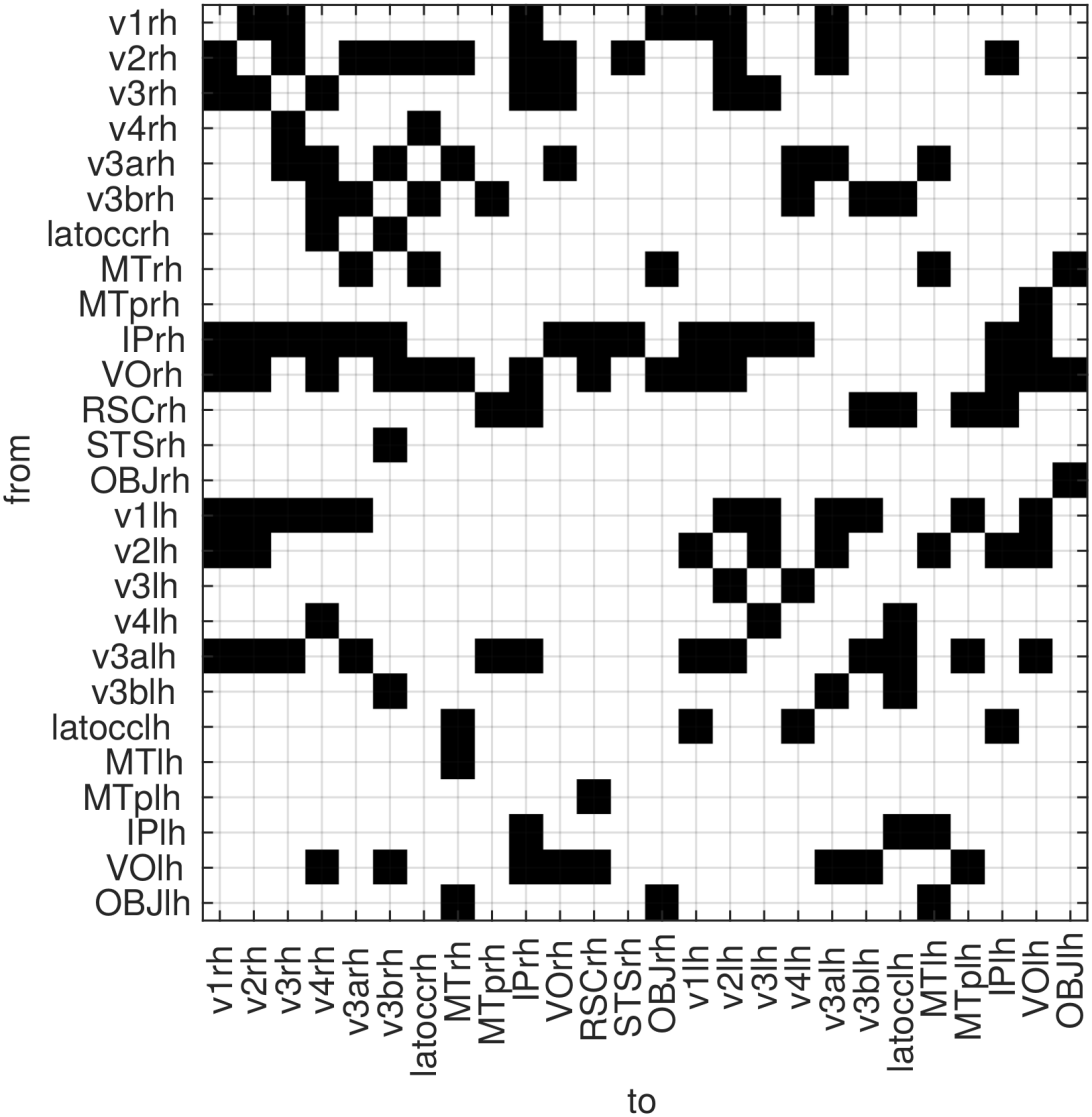
The causal configuration matrix computed on the empirical fMRI data, the black squares indicate a causal interaction from the rows to the columns.

Finally, we report the causal configuration matrix that is obtained by the predictions of GMEP. Based on our findings, we adopt the lag-independent prior associated with the hemisphere partition. The configuration matrix is computed by following the same approach that was used in the synthetic data. About the number of time points, the same proportion of elements in the design matrix and unknowns was also preserved for the empirical data. Thus, since in this dataset *d*_*y*_ = 26, to be consistent with the previous analyses, 1638 time points were selected for the inference. The causal configuration matrix is shown in Fig 8 and it contains a black square when a causal interaction is determined from a region along the rows to a region along the columns. Based on this matrix, we tested the significance of the sum of the overlapping connections between the two hemispheres, i.e. the intersection of the two sets of connections within hemisphere. And the significance of the sum of the connections of homologous areas across hemispheres. In both cases, the null hypothesis was rejected with a significance level of 0.01. The distribution of the null hypothesis was computed by randomly permuting the estimated connections for 1 million of iterations.

## Discussion

In this paper we analysed a novel approach for Bayesian linear modelling with structured prior (GMEP) in the context of the MAR identification with the aim to apply it for a Granger-based estimate of directed functional brain connectivity.

We first made a simple comparison with other standard linear estimators to see how GMEP is placed in relation to them. By evaluating the NRMSE of the coefficient estimates, GMEP showed the most accurate predictions. Our results also provide an insight into how the connection density and the dimensionality influence the inferences. In particular, we obtained that given a certain dimensionality, the complexity of the estimation problem increases with the increase in connection density. It is important to highlight that dimensionality and number of unknowns (coefficients) are related, thus in all of our experiments the proportion between number of elements in the design matrix and unknowns was kept constant across datasets.

One of the main advantages of GMEP is its flexibility in the definition of the structured prior. Thus this aspect was studied through several simulations. The simulations were meant to test how the structured prior affected the predictions under different conditions of dimensionality and connection density. In the case of sparse datasets, i.e. datasets with low connection density, modelling the sparsity improves the performance of GMEP.

We modelled the sparsity by two types of structured priors. That is, the ARD prior and the lag-independent prior, which were compared with the uniform Gaussian prior.

The uniform Gaussian and the ARD priors can be seen as two extreme scenarios in terms of model complexity. In the case of the uniform Gaussian prior, the model complexity is very low since all the coefficients that are involved in the modelling of the same time series are clustered in the same group. Thus they are supposed to be drawn from the same distribution, i.e. they are assumed to have the same sparsity. This assumption is realistic only in case of very high connection density. Indeed, under this condition the uniform Gaussian and the lag-independent priors behave similarly. On the other hand, the ARD prior models the sparsity structure very accurately by assigning a single group to each coefficient. Even though, theoretically it should be able to properly model the real sparsity of the coefficients, in practice it is beneficial only in case of very sparse interactions. The drawback of the high complexity of the ARD prior is clearly shown in the Figs 4 and 5 where it appears that ARD overfits the training data.

The lag-independent prior was shown to always outperform the other priors or, in the worst case, be equal to the uniform Gaussian prior. This result was expected since such a prior models the actual sparsity structure of the coefficients, forming an optimal compromise in term of model complexity. Summarizing, these results provide evidence of the importance of adding prior knowledge about the sparsity structure of the coefficients in the model.

Regarding the ability to predict the causal interactions among time series, we can conclude that GMEP reaches a balanced accuracy that is the 10% higher than the one of MVGC for some levels of noise. This result is important for the application in empirical settings in which we do not know neither the true amount of noise nor the true connection density. Even though the experiment was restricted to just three dimensions and a fixed number of time points, it shows that GMEP can provide meaningful advantages, particularly for medium noise levels.

The experiments on the empirical data under the three structured priors showed that, in agreement with the simulations, the lag-independent prior performs consistently better under different data lengths. This evidence suggests that the assumption of time independence of the causal configuration, is more plausible than assuming a shared or completely independent sparsity structure. Moreover, the improvement given by the inclusion of the hemisphere partitioning in the structured prior, confirms our assumption that the sparsity structure of the coefficients reflects the hemisphere structure. Regarding the causal configuration matrix, the simple statistical tests that were run on it, suggest significant symmetries on the connections within and across hemispheres.

## Conclusion

A new Bayesian method for linear regression with structured prior (GMEP) was proposed and applied in the context of the MAR identification. The purpose was to identify the MAR model in order to obtain a Granger-based estimate of the causal configuration matrix from a given set of time series. The main advantage of GMEP is a flexible definition of various structured priors associated with the sparsity structure of the MAR coefficients. We investigated GMEP among standard linear estimators on simulated datasets with different dimensionalities and connection densities. Moreover, we focused on the effect of defining different structured priors. And we showed the benefit of including information on the sparsity structure of the coefficients in their prior definition. In the same simulation framework, we identified under with conditions the inference of the causal configuration matrices performed by GMEP achieves better results than the inference done by a standard Granger toolbox (MVGC). Finally, we reported a simple example with empirical fMRI data showing that the enrichment of the structured prior by the inclusion of anatomical information i.e. the hemisphere partitioning, leads to a better inference.

As future works, regarding the characterization of GMEP, we will focus on the effect of the structured prior when it is not coherent with the modelling assumption. Moreover, we consider of interest also a comparison with other Bayesian methods with group sparsity prior. About the application on fMRI data, the effect of the hemodynamic response on Granger-based methods has always been a source of debate thus further investigations will be devoted in order to study how it affects the results of GMEP.

## Supporting information

S1 Supplementary Materials An expectation propagation approach for generalized linear models with hierarchical priors. (PDF)
