# Supplementary material for: Bayesian estimation of directed functional coupling from brain recordings
Source: PLoS One. 2017 May 18;12(5):e0177359. doi: 10.1371/journal.pone.0177359 (PMC5436686; doi:10.1371/journal.pone.0177359)
Supplement: S1 Supplementary Materials — (PDF) [file pone.0177359.s001.pdf]

Supplementary materials

1 An Expectation Propagation Approach for Generalized Linear Models with Hierarchical Priors

This section describes a novel approach for hierarchical linear models that enables flexible definition of various non-conjugate observations models and coefficient priors using hyperparameters with a multivariate Gaussian prior. Approximate posterior inference is done using expectation propagation for both the linear coefficients and the hyperparameters.

1.1 Model Description

We consider generalized linear models with multiple output variables, where the probability density of the observed $d_y \times 1$ output vectors $\mathbf{y}_i$ depend on the $d_x \times 1$ input vectors $\mathbf{x}_i$ through a linear transformation $\mathbf{W}^{\mathrm{T}}\mathbf{x}_i$. We assume that given n input-output pairs denoted by $\mathcal{D} = \{\mathbf{x}_i, \mathbf{y}_i\}_{i=1}^{n}$ the observation model can be written as

$$p(\mathbf{Y}|\mathbf{X}\mathbf{W}, \boldsymbol{\theta}) = \prod_{i=1}^{n} p(\mathbf{y}_i|\mathbf{W}^{\mathrm{T}}\mathbf{x}_i, \mathbf{V}_i^{\mathrm{T}}\boldsymbol{\theta}), \tag{1}$$

where $\mathbf{Y} = [\mathbf{y}_1, ..., \mathbf{y}_n]^{\mathrm{T}}$ is a $n \times d_y$ output variable matrix, $\mathbf{X} = [\mathbf{x}_1, ..., \mathbf{x}_n]^{\mathrm{T}}$ a $n \times d_x$ input variable matrix, and $\mathbf{W} = [\mathbf{w}_1, ..., \mathbf{w}_{d_y}]$ a $d_x \times d_y$ coefficient matrix. Each of the n likelihood terms depends on the $d_\theta \times 1$ hyperparameter vector $\boldsymbol{\theta}$ via a known transformation matrix $\mathbf{V}_i$. For the inference algorithm to remain computationally feasible, we assume that in typical applications each likelihood term depends only on a low-dimensional transformed variable $\mathbf{z}_i = \mathbf{V}_i^{\mathrm{T}}\boldsymbol{\theta}$.

We form a hierarchical prior family for the linear coefficients by combining them with the hyperparameters using known linear transformations. We assume that the prior for the coefficients $\mathbf{W}$ can be factored into m terms according to

$$p(\mathbf{W}|\boldsymbol{\theta}) = \prod_{j=n+1}^{n+m} t_j(\mathbf{U}_j^{\mathrm{T}}\mathbf{w}, \mathbf{V}_j^{\mathrm{T}}\boldsymbol{\theta}), \tag{2}$$

where $\mathbf{w} = \mathrm{vec}(\mathbf{W}) = [\mathbf{w}_1^{\mathrm{T}}, ..., \mathbf{w}_{d_y}^{\mathrm{T}}]^{\mathrm{T}}$, and the transformation matrices $\mathbf{U}_j$ and $\mathbf{V}_j$ are known. We choose a multivariate Gaussian prior distributions for the

hyperparameters $\boldsymbol{\theta}$:

$$p(\boldsymbol{\theta}) = \mathcal{N}(\boldsymbol{\mu}_{\theta,0}, \boldsymbol{\Sigma}_{\theta,0}), \tag{3}$$

where $\boldsymbol{\mu}_{\theta,0}$ is the prior mean vector and $\boldsymbol{\Sigma}_{\theta,0}$ the prior covariance matrix.

Combining the likelihood (1) with the priors (2) and (3), gives the following posterior distribution

$$p(\mathbf{w}|\mathcal{D}) = Z^{-1} \prod_{i=1}^{n} p(\mathbf{y}_i|\mathbf{W}^{\mathrm{T}}\mathbf{x}_i, \mathbf{V}_i^{\mathrm{T}}\boldsymbol{\theta}) \prod_{j=n+1}^{n+m} t_j(\mathbf{U}_j^{\mathrm{T}}\mathbf{w}, \mathbf{V}_j^{\mathrm{T}}\boldsymbol{\theta}) p(\boldsymbol{\theta}), \tag{4}$$

where $Z = p(\mathbf{Y}|\mathbf{X}) = \int p(\mathbf{Y}|\mathbf{W}, \boldsymbol{\theta}, \mathbf{X}) p(\mathbf{W}|\boldsymbol{\theta}) p(\boldsymbol{\theta}) d\mathbf{W} d\boldsymbol{\theta}$ is the marginal likelihood or the model evidence. The general model definition of equations (1)–(3) enables implementation of various different linear models via the choice of transformations $\{\mathbf{U}_i\}_{i=n+1}^{n+m}$ and $\{\mathbf{V}_i\}_{i=1}^{n+m}$, and in the following we summarize some common model specifications.

1.1.1 Regression Models

A Gaussian observation model with unknown noise variances for each output $k = 1, ..., d_y$ can be formed as

$$\begin{aligned} p(\mathbf{y}_i|\mathbf{W}^{\mathrm{T}}\mathbf{x}_i, \mathbf{V}_i^{\mathrm{T}}\boldsymbol{\theta}) &= \mathcal{N}\Big(\mathbf{y}_i|\mathbf{W}^{\mathrm{T}}\mathbf{x}_i, \mathrm{diag}\left(\exp(\mathbf{V}_i^{\mathrm{T}}\boldsymbol{\theta})\right)\Big) \\ &= \prod_{k=1}^{d_y} \mathcal{N}\big(y_{i,j}|\mathbf{w}_k^{\mathrm{T}}\mathbf{x}_i, \exp(\mathbf{v}_{i,k}^{\mathrm{T}}\boldsymbol{\theta})\big), \end{aligned} \tag{5}$$

where the exponentiation is taken component-wise and $\mathbf{V}_i = [\mathbf{v}_{i,1}, ..., \mathbf{v}_{i,d_y}]$ is a $d_\theta \times d_y$ matrix that encodes our noise model. For example, a common noise level for all outputs can be obtained by setting $\mathbf{V}_i = \mathbf{1}_{d_y}^{\mathrm{T}}$. Alternatively, independent noise levels for all outputs are obtained by choosing $\mathbf{V}_i = \mathbf{I}_{d_y}$, which results in d_y independent regression problems unless the coefficient are not coupled through the prior $p(\mathbf{W}|\boldsymbol{\theta})$.

An outlier robust observation model could be formed, e.g., by setting choosing $\mathbf{V}_i = \mathbf{e}_i \mathbf{1}_{d_y}^{\mathrm{T}}$ in equation (5), where $\mathbf{e}_i$ is the i:th n-dimensional unit vector. This model assumes that a potential outlier corrupts all components of $\mathbf{y}_i$, and it corresponds to a normal scale-mixture model with a log-normal mixing distribution. A heavy-tailed model could also be obtained by replacing the Gaussian distribution in (5) with a Laplace distribution or a Student-t distribution and using the same noise level hyperparameter for all observations.

1.1.2 Classification Models

A binary classifier can by formed by choosing

$$p(\mathbf{y}_i|\mathbf{W}^{\mathrm{T}}\mathbf{x}_i, \mathbf{V}_i^{\mathrm{T}}\boldsymbol{\theta}) = \prod_{k=1}^{d_y} \Phi(y_{i,k}\mathbf{w}_k^{\mathrm{T}}\mathbf{x}_i), \tag{6}$$

where $y_i \in [-1, 1]$ is the i:th class label and $\Phi(x)$ is the logistic or the Gaussian cumulative distribution function. In this case there are no hyperparameters $\boldsymbol{\theta}$ associated with the likelihood terms. This observation model results in d_y independent regression problems unless the coefficient are not coupled through the prior $p(\mathbf{W}|\boldsymbol{\theta})$.

A multi-class classifier can be formed by using the multinomial probit observation model, which can be written as

$$p(y_i|\mathbf{W}^{\mathrm{T}}\mathbf{x}_i, \mathbf{V}_i^{\mathrm{T}}\boldsymbol{\theta}) = \int \prod_{k=1,k\neq y_i}^{d_y} \mathcal{N}(u_i|0,1)\Phi(u_i + \mathbf{w}_{y_i}^{\mathrm{T}}\mathbf{x}_i - \mathbf{w}_k^{\mathrm{T}}\mathbf{x}_i)du_i, \tag{7}$$

where $y_i \in \{1, .., d_y\}$ is the class label and the integration over the nuisance variable u_i ensures that the probabilities for all class assignments of y_i sum to one. The EP inference approach for Gaussian process classifiers described by Riihimäki et al. [9] can be readily applied for this type of likelihood terms.

1.1.3 Scale-Mixture Coefficient Priors

Various types of scale-mixture coefficient priors can be formed by using Gaussian distributions in (2):

$$\begin{aligned} p(\mathbf{W}|\boldsymbol{\theta}) &= Z(\boldsymbol{\theta})^{-1} \prod_{j=n+1}^{n+m} \exp\left(-\frac{1}{2}\exp(-\mathbf{V}_j^{\mathrm{T}}\boldsymbol{\theta})(\mathbf{w}^{\mathrm{T}}\mathbf{U}_j)^2\right) \\ &\propto \prod_{j=n+1}^{n+m} \mathcal{N}\Big(\mathbf{U}_j^{\mathrm{T}}\mathbf{w}|0, \exp\left(\mathbf{V}_j^{\mathrm{T}}\boldsymbol{\theta}\right)\Big) \end{aligned} \tag{8}$$

where transformation $\mathbf{U}_j^{\mathrm{T}}\mathbf{w}$ and $\mathbf{V}_j^{\mathrm{T}}\boldsymbol{\theta}$ are assumed to yield scalar random variables and the normalization term is given by

$$\log Z(\boldsymbol{\theta}) = \frac{d_x}{2}\log(2\pi) - \frac{1}{2}\log|\mathbf{Q}|, \quad \text{where} \quad \mathbf{Q} = \sum_{j=1}^{n+m} \mathbf{U}_j \exp(-\mathbf{V}_j^{\mathrm{T}}\boldsymbol{\theta})\mathbf{U}_j^{\mathrm{T}}.$$

To keep the prior factorizable, we assume that the vectors $\mathbf{U}_1, ..., \mathbf{U}_m$ are orthonormal, because then $\lambda_j = \exp(-\mathbf{V}_j^{\mathrm{T}}\boldsymbol{\theta})$ are the eigenvalues of $\mathbf{Q}$ and the normalization term reduces to $\log Z(\boldsymbol{\theta}) = \frac{1}{2}(d_w \log(2\pi) + \sum_j \mathbf{V}_j^{\mathrm{T}}\boldsymbol{\theta})$, where $d_w = d_x d_y$ is the dimension of $\mathbf{w} = \mathrm{vec}(\mathbf{W})$. In the standard setting when $\mathbf{U}_j = \mathbf{e}_j$, the components of $\mathbf{w}$ are conditionally independent given $\boldsymbol{\theta}$. With general transformation vectors $\mathbf{U}_1, ..., \mathbf{U}_m$, we could either use an un-normalized prior by using $\log Z(\boldsymbol{\theta}) \approx \frac{1}{2}(m \log(2\pi) + \sum_j \mathbf{V}_j^{\mathrm{T}}\boldsymbol{\theta})$ or constrain the number of different hyperparameter transformations $\mathbf{V}_j$ to a low number so that $|\mathbf{Q}|$ can be evaluated efficiently.

Using (8) we can implement various types of commonly used coefficient priors:

- **A uniform Gaussian** prior with an unknown scalar prior variance (similar to ridge regression) can be obtained by choosing $\mathbf{U}_j = \mathbf{e}_j$ $(d_w \times 1)$ and $\mathbf{V}_j = 1$ for $j = n+1, ..., n+d_w$. This prior assumes that all the d_y different outputs share the same prior variance hyperparameter.

  We could also set different variance hyperparameters for each output by choosing $\mathbf{V}_j = \mathbf{e}_k$ $(d_y \times 1)$ for $j = (k-1)d_x + n + 1, ..., (k-1)d_x + n + d_x$ and $k = 1, .., d_y$. Note that this leads to d_y different inference problems, if the coefficients related to different outputs are not coupled through the observation model.

- **Automatic relevance determination (ARD)** prior can be formed by assigning individual scale hyperparameters to the coefficients related to each input variable. One option is to set $\mathbf{U}_j = \mathbf{e}_j$ $(d_w \times 1)$ and $\mathbf{V}_j = \mathbf{e}_l$ $(d_x \times 1)$ with $j = k - 1 + l + n$ for $l = 1, ..., d_x$ and $k = 1, ..., d_y$. This construction assumes that all outputs share the same sparsity structure, i.e., a coefficient gets a large prior variance if the corresponding coefficients from the other outputs are also large.

  Alternatively individual scale parameters could be assigned to all coefficients but this would result in a much larger number of hyperparameters and no information sharing between the outputs.

- **Group sparsity** priors can be constructed by defining possibly overlapping groups as $\mathbf{U}_j = \mathbf{e}_j$ $(d_w \times 1)$ and $\mathbf{V}_j = [1, 0, 1, 0, 0, ..., 0]^{\mathrm{T}}$ $(n_g \times 1)$, where $\mathbf{V}_j$ is a binary vector indicating the group memberships for each coefficient and n_g is the number of different groups. The groups could be defined either so that they combine coefficients from different output units into same groups or completely separately for each output.

The previous prior constructions could be implemented also with other type of univariate distributions in place of Gaussian in (8). For example, we could use more heavy-tailed alternatives such as the Laplace distribution but then it would be more sensible to use only uniform scale parameters that control the overall magnitude.

1.1.4 Gaussian Process Priors

Instead of sparsity-promoting priors we can also use Gaussian process (GP) priors in equation (8) and there are at least two distinct approaches to this [for an introduction to GP models, see 8]. One option is to set a GP prior directly to the coefficients $\mathbf{w}$:

$$p(\mathbf{W}|\boldsymbol{\theta}) = t_{n+1}(\mathbf{U}_j^{\mathrm{T}}\mathbf{w}, \mathbf{V}_j^{\mathrm{T}}\boldsymbol{\theta}) = \mathcal{N}\big(\mathbf{w}|\mathbf{0}, \mathbf{K}(\boldsymbol{\theta}_{\mathrm{GP}}, \mathbf{r}, \mathbf{t})\big), \tag{9}$$

where $\mathbf{K}(\boldsymbol{\theta}_{\mathrm{GP}}, \mathbf{r}, \mathbf{t})$ is the prior covariance matrix that encodes, e.g., smoothness assumptions between the coefficients associated with different spatial and temporal coordinates denoted by $\mathbf{r}$ and $\mathbf{t}$ respectively. Inference on the GP hyperparameters $\boldsymbol{\theta}_{\mathrm{GP}}$, which control e.g. the smoothness properties induced

by the prior, is challenging due to the complex nonlinear dependency of $\mathbf{w}$ and $\boldsymbol{\theta}_{\mathrm{GP}}$ through the chosen covariance function [8]. Therefore, following the usual practice with GP models, maximum marginal posterior (MAP) estimates could be obtained via gradient-based optimization using an approximation to the marginal likelihood

$$Z(\boldsymbol{\theta}_{\mathrm{GP}}) = \int p(\mathbf{Y}|\mathbf{W}, \boldsymbol{\theta}_{\mathrm{L}}, \mathbf{X}) p(\mathbf{W}|\boldsymbol{\theta}_{\mathrm{GP}}) p(\boldsymbol{\theta}_{\mathrm{L}}) d\mathbf{W} d\boldsymbol{\theta}_{\mathrm{L}}.$$

The other approach for utilizing Gaussian processes would be to place a GP prior on the hyperparameters $\boldsymbol{\theta}$ by redefining (3) as

$$p(\boldsymbol{\theta}) = \mathcal{N}(\mathbf{0}, \mathbf{K}(\boldsymbol{\theta}_{\mathrm{GP}}, \mathbf{r}, \mathbf{t})), \tag{10}$$

where $\mathbf{K}(\boldsymbol{\theta}_{\mathrm{GP}}, \mathbf{r}, \mathbf{t})$ is a prior covariance matrix that encodes, e.g., the smoothness assumptions between the scale hyperparmeters associated with different spatial and temporal coordinates denoted by $\mathbf{r}$ and $\mathbf{t}$ respectively. The main conceptual difference with the GP prior for $\mathbf{w}$ is that the signs of the coefficients can still vary in nearby locations but the sparsity structure is assumed to be smooth. In this setting, inference on $\mathbf{w}$ and $\boldsymbol{\theta}$ can be done using EP, whereas MAP estimates could be determined for the GP hyperparameters $\boldsymbol{\theta}_{\mathrm{GP}}$. Similar approaches based on GPs and Gaussian markov random fields in combination with spike and slab priors have been proposed by Andersen et al. [1] and Miguel Hernández-Lobato et al. [4].

1.2 Approximate Inference

This section describes an EP algorithm that is used to form a deterministic Gaussian approximation to the posterior distribution (4).

1.2.1 Posterior Approximation

To simplify the notation, we first denote $\mathbf{W}^{\mathrm{T}}\mathbf{x}_i = \mathbf{U}_i^{\mathrm{T}}\mathbf{w}$ for $i = 1, ..., n$, where $\mathbf{U}_i = \mathbf{I}_{d_y} \otimes \mathbf{x}_i$, and then write the posterior distribution (4) as

$$p(\mathbf{w}, \boldsymbol{\theta}|\mathcal{D}) = Z^{-1} \prod_{i=1}^{n+m} t_i(\mathbf{U}_i^{\mathrm{T}}\mathbf{w}, \mathbf{V}_i^{\mathrm{T}}\boldsymbol{\theta}) p(\boldsymbol{\theta}), \tag{11}$$

where $t_i(\cdot)$ are some known site functions. We use expectation propagation [7] to approximate the posterior distribution (11) with

$$p(\mathbf{w}, \boldsymbol{\theta}|\mathcal{D}) \approx Z_{\mathrm{EP}}^{-1} \prod_{i=1}^{n+m} \tilde{Z}\tilde{t}_{\mathbf{w},i}(\mathbf{w})\tilde{t}_{\boldsymbol{\theta},i}(\boldsymbol{\theta}) p(\boldsymbol{\theta}) = q(\mathbf{w})q(\boldsymbol{\theta}), \tag{12}$$

where each analytically intractable site function is approximated with a tractable site approximation that can be factored between $\mathbf{w}$ and $\boldsymbol{\theta}$:

$$t_i(\mathbf{U}_i^{\mathrm{T}}\mathbf{w}, \mathbf{V}_i^{\mathrm{T}}\boldsymbol{\theta}) \approx \tilde{t}_i(\mathbf{w}, \boldsymbol{\theta}) = \tilde{Z}_i\tilde{t}_{\mathbf{w},i}(\mathbf{w})\tilde{t}_{\boldsymbol{\theta},i}(\boldsymbol{\theta}), \tag{13}$$

and $\tilde{Z}_i$ is a scalar scaling parameter, which is needed to form an EP approximation for the marginal likelihood of the observed data $Z = p(\mathbf{Y}|\mathbf{X}) \approx Z_{\mathrm{EP}}$.

We assume Gaussian site approximations of the form

$$\begin{aligned} \tilde{t}_{\mathbf{w},i}(\mathbf{w}) &= \psi\left(\mathbf{w}^{\mathrm{T}}\mathbf{U}_i, \tilde{\boldsymbol{\nu}}_{\mathbf{w},i}, \tilde{\mathbf{T}}_{\mathbf{w},i}\right) \\ \tilde{t}_{\boldsymbol{\theta},i}(\boldsymbol{\theta}) &= \psi\left(\boldsymbol{\theta}^{\mathrm{T}}\mathbf{V}_i, \tilde{\boldsymbol{\nu}}_{\boldsymbol{\theta},i}, \tilde{\mathbf{T}}_{\boldsymbol{\theta},i}\right) \end{aligned} \tag{14}$$

where $\tilde{\boldsymbol{\nu}}_{\mathbf{w},i}$ and $\tilde{\mathbf{T}}_{\mathbf{w},i}$ are the site location and precision parameters for $\mathbf{w}$, $\tilde{\boldsymbol{\nu}}_{\boldsymbol{\theta},i}$ and $\tilde{\mathbf{T}}_{\boldsymbol{\theta},i}$ are the corresponding site parameters for $\boldsymbol{\theta}$, and the un-normalized Gaussian site term approximations are defined using

$$\psi(\mathbf{w}, \mathbf{h}, \mathbf{Q}) = \exp\left(-\frac{1}{2}\mathbf{w}^{\mathrm{T}}\mathbf{Q}\mathbf{w} + \mathbf{h}^{\mathrm{T}}\mathbf{w}\right). \tag{15}$$

The dimension of the site parameters are defined by the corresponding transformation matrices, e.g., $\tilde{\mathbf{T}}_{\mathbf{w},i}$ is a $d_i \times d_i$ matrix and $\tilde{\boldsymbol{\nu}}_{\mathbf{w},i}$ is a $d_i \times 1$ vector if $\mathbf{U}_i$ has d_i columns.

Multiplying the site approximations (14) together according to (12) gives the following Gaussian approximations for $\mathbf{w}$ and $\boldsymbol{\theta}$:

$$\begin{aligned} q(\mathbf{w}) &= Z(\mathbf{h}_{\mathbf{w}}, \mathbf{Q}_{\mathbf{w}})^{-1}\psi(\mathbf{w}, \mathbf{h}_{\mathbf{w}}, \mathbf{Q}_{\mathbf{w}}) = \mathcal{N}(\mathbf{w}|\boldsymbol{\mu}_{\mathbf{w}}, \boldsymbol{\Sigma}_{\mathbf{w}}) \\ q(\boldsymbol{\theta}) &= Z(\mathbf{h}_{\boldsymbol{\theta}}, \mathbf{Q}_{\boldsymbol{\theta}})^{-1}\psi(\mathbf{w}, \mathbf{h}_{\boldsymbol{\theta}}, \mathbf{Q}_{\boldsymbol{\theta}}) = \mathcal{N}(\mathbf{w}|\boldsymbol{\mu}_{\boldsymbol{\theta}}, \boldsymbol{\Sigma}_{\boldsymbol{\theta}}), \end{aligned} \tag{16}$$

where the moment and natural parameters are related by $\boldsymbol{\mu}_{\mathbf{w}} = \mathbf{Q}_{\mathbf{w}}^{-1}\mathbf{h}_{\mathbf{w}}$ and $\boldsymbol{\Sigma}_{\mathbf{w}} = \mathbf{Q}_{\mathbf{w}}^{-1}$ (and analogously for $\boldsymbol{\theta}$), the natural parameters are given by

$$\begin{aligned} \mathbf{h}_{\mathbf{w}} &= \sum_{i=1}^{n+m} \mathbf{U}_i\tilde{\boldsymbol{\nu}}_{\mathbf{w},i} & \mathbf{Q}_{\mathbf{w}} &= \sum_{i=1}^{n+m} \mathbf{U}_i\tilde{\mathbf{T}}_{\mathbf{w},i}\mathbf{U}_i^{\mathrm{T}} \\ \mathbf{h}_{\boldsymbol{\theta}} &= \sum_{i=1}^{n+m} \mathbf{V}_i\tilde{\boldsymbol{\nu}}_{\boldsymbol{\theta},i} + \boldsymbol{\Sigma}_{\boldsymbol{\theta},0}^{-1}\boldsymbol{\mu}_{\boldsymbol{\theta},0} & \mathbf{Q}_{\boldsymbol{\theta}} &= \sum_{i=1}^{n+m} \mathbf{V}_i\tilde{\mathbf{T}}_{\boldsymbol{\theta},i}\mathbf{V}_i^{\mathrm{T}} + \boldsymbol{\Sigma}_{\boldsymbol{\theta},0}^{-1}, \end{aligned} \tag{17}$$

and the normalization factor (or the partition function) is defined as

$$\log Z(\mathbf{h}, \mathbf{Q}) = \log \int \psi(\mathbf{w}, \mathbf{h}, \mathbf{Q}) d\mathbf{w} = \frac{d}{2}\log 2\pi - \frac{1}{2}\log|\mathbf{Q}| + \frac{1}{2}\mathbf{h}^{\mathrm{T}}\mathbf{Q}^{-1}\mathbf{h}. \tag{18}$$

1.2.2 Expectation Propagation

The standard EP algorithm [7] updates the parameters of the site approximations and the posterior approximation $q(\mathbf{w}, \boldsymbol{\theta})$ sequentially. At each iteration, first a proportion η of the i:th site term is removed from the posterior approximation to obtain a cavity distribution:

$$q_{-i}(\mathbf{w}, \boldsymbol{\theta}) = q_{-i}(\mathbf{w})q_{-i}(\boldsymbol{\theta}) \propto q(\mathbf{w})q(\boldsymbol{\theta})\tilde{t}_{\mathbf{w},i}(\mathbf{w})^{-\eta}\tilde{t}_{\boldsymbol{\theta},i}(\boldsymbol{\theta})^{-\eta}, \tag{19}$$

where $\eta \in (0, 1]$ is a fraction parameter that can be adjusted to implement fractional (or power) EP updates [5, 6]. Then, the i:th site approximation is replaced with the exact site term to form a tilted distribution

$$\hat{p}_i(\mathbf{w}, \boldsymbol{\theta}) = \hat{Z}_i^{-1} q_{-i}(\mathbf{w}, \boldsymbol{\theta}) t_i(\mathbf{U}_i^{\mathrm{T}}\mathbf{w}, \mathbf{V}_i^{\mathrm{T}}\boldsymbol{\theta})^{\eta}, \tag{20}$$

where $\hat{Z}_i = \int q_{-i}(\mathbf{w}, \boldsymbol{\theta}) t_i(\mathbf{U}_i^{\mathrm{T}}\mathbf{w}, \mathbf{V}_i^{\mathrm{T}}\boldsymbol{\theta})^{\eta} d\mathbf{w} d\boldsymbol{\theta}$ is a normalization factor, which in case $i \leq n$ can also be thought of as an approximation to the leave-one-out (LOO) predictive density of the excluded data point $\mathbf{y}_i$. The tilted distribution can be regarded as a more refined approximation to the true posterior distribution. Next, the algorithm attempts to match the approximate posterior distribution $q(\mathbf{w}, \boldsymbol{\theta})$ with $\hat{p}_i(\mathbf{w}, \boldsymbol{\theta})$ by finding a member of the chosen approximate family $\hat{q}_i(\mathbf{w}, \boldsymbol{\theta})$ that satisfies

$$\hat{q}_i(\mathbf{w}, \boldsymbol{\theta}) = \arg\min_{q_i} \mathrm{KL}\left(\hat{p}_i(\mathbf{w}, \boldsymbol{\theta}) || q_i(\mathbf{w}, \boldsymbol{\theta})\right), \tag{21}$$

where KL denotes the Kullback-Leibler divergence and q_i can be factored as $q_i(\mathbf{w}, \boldsymbol{\theta}) = q_i(\mathbf{w}) q_i(\boldsymbol{\theta})$. Then, the parameters of the i:th site terms are updated so that the moments of $q(\mathbf{w}, \boldsymbol{\theta})$ match with $\hat{q}_i(\mathbf{w}, \boldsymbol{\theta})$:

$$\hat{q}_i(\mathbf{w}, \boldsymbol{\theta}) \equiv q(\mathbf{w}, \boldsymbol{\theta}) \propto q_{-i}(\mathbf{w}) q_{-i}(\boldsymbol{\theta}) \tilde{t}_{\mathbf{w},i}(\mathbf{w})^{\eta} \tilde{t}_{\boldsymbol{\theta},i}(\boldsymbol{\theta})^{\eta}. \tag{22}$$

Finally, the posterior approximation $q(\mathbf{w}, \boldsymbol{\theta})$ is updated according to the changes in the site parameters. These steps are repeated for all sites in some suitable order until convergence.

From now on, we refer to the previously described EP update scheme, where the posterior approximations $q(\mathbf{w})$ and $q(\boldsymbol{\theta})$ are refreshed after each of the $n+m$ site updates, as sequential EP. If the posterior approximations are updated only after new site parameter values have been determined for all the n likelihood sites or m prior sites, we refer to parallel EP [see, e.g., 2].

1.2.3 Algorithm Description

With the chosen model structure and approximate family, the EP algorithm can be implemented as follows. First, initialize the site parameters, for example, as $\tilde{\boldsymbol{\nu}}_{\mathbf{w},i} = \mathbf{0}$, $\{\tilde{\mathbf{T}}_{\mathbf{w},i} = \mathbf{0}\}_{i=1}^{n}$, $\{\tilde{\mathbf{T}}_{\mathbf{w},i} = \alpha\mathbf{I}\}_{i=n+1}^{m}$, $\tilde{\boldsymbol{\nu}}_{\boldsymbol{\theta},i} = \mathbf{0}$, and $\tilde{\mathbf{T}}_{\boldsymbol{\theta},i} = \mathbf{0}$, where α is an initial uniform prior variance for the coefficients, and compute the approximations $q(\mathbf{w})$ and $q(\boldsymbol{\theta})$. Then iterate the following steps at a chosen order for each $i = 1, ..., n+m$:

1. Determine the parameters of the cavity distribution (19). Compute first the mean $\boldsymbol{\mu}_i$ and the covariance $\boldsymbol{\Sigma}_i$ of the approximate marginal distribution of the transformed variable $\mathbf{z}_i = \mathbf{U}_i^{\mathrm{T}}\boldsymbol{\mu}_{\mathbf{w}}$: $q(\mathbf{z}_i) = \mathcal{N}(\boldsymbol{\mu}_i, \boldsymbol{\Sigma}_i)$, where $\boldsymbol{\mu}_i = \mathbf{U}_i^{\mathrm{T}}\boldsymbol{\mu}_{\mathbf{w}}$ and $\boldsymbol{\Sigma}_i = \mathbf{U}_i^{\mathrm{T}}\boldsymbol{\Sigma}_{\mathbf{w}}\mathbf{U}_i$. The cavity distribution for the transformed variable $\mathbf{z}_i$ is then given by $q_{-i}(\mathbf{z}_i) = \mathcal{N}(\boldsymbol{\mu}_{-i}, \boldsymbol{\Sigma}_{-i})$, where

$$\begin{aligned} \boldsymbol{\mu}_{-i} &= \boldsymbol{\Sigma}_{-i}(\boldsymbol{\Sigma}_i^{-1}\boldsymbol{\mu}_i - \eta\tilde{\boldsymbol{\nu}}_{\mathbf{w},i}) \\ \boldsymbol{\Sigma}_{-i} &= \left(\boldsymbol{\Sigma}_i^{-1} - \eta\tilde{\mathbf{T}}_{\mathbf{w},i}\right)^{-1}. \end{aligned} \tag{23}$$

Compute the cavity distribution $q_{-i}(\boldsymbol{\phi}_i)$ for the transformed variable $\boldsymbol{\phi}_i = \mathbf{V}_i^{\mathrm{T}}\boldsymbol{\theta}$ analogously.

2. Compute the sufficient statistics with respect to the i:th tilted distribution (21). For the the transformed variables $\mathbf{z}_i$ and $\boldsymbol{\phi}_i$, determine (or approximate) the marginal means $\hat{\boldsymbol{\mu}}_i = \mathrm{E}_{\hat{p}_i}(\mathbf{z}_i)$ and $\hat{\boldsymbol{\mu}}_{\boldsymbol{\phi},i} = \mathrm{E}_{\hat{p}_i}(\boldsymbol{\phi}_i)$ and covariances $\hat{\boldsymbol{\Sigma}}_i = \mathrm{E}_{\hat{p}_i}(\mathbf{z}_i\mathbf{z}_i^{\mathrm{T}}) - \hat{\boldsymbol{\mu}}_i\hat{\boldsymbol{\mu}}_i^{\mathrm{T}}$ and $\hat{\boldsymbol{\Sigma}}_{\boldsymbol{\phi},i} = \mathrm{E}_{\hat{p}_i}(\boldsymbol{\phi}_i\boldsymbol{\phi}_i^{\mathrm{T}}) - \hat{\boldsymbol{\mu}}_{\boldsymbol{\phi},i}\hat{\boldsymbol{\mu}}_{\boldsymbol{\phi},i}^{\mathrm{T}}$ with respect to the the marginal tilted distribution:
$$\hat{p}_i(\mathbf{z}_i, \boldsymbol{\phi}_i) = \hat{Z}_i^{-1} t_i(\mathbf{z}_i, \boldsymbol{\phi}_i)^{\eta} q_{-i}(\mathbf{z}_i) q_{-i}(\boldsymbol{\phi}_i). \tag{24}$$
If the normalization term $\hat{Z}_i$ can be computed analytically, the sufficient statistics can be determined by differentiating $\log \hat{Z}_i$ with respect to the cavity parameters, e.g., wrt. $\boldsymbol{\Sigma}_{-i}^{-1}\boldsymbol{\mu}_{-i}$ and $\boldsymbol{\Sigma}_{-i}^{-1}$ for $\mathbf{z}_i$. Otherwise, the necessary moments have to approximated with a suitable method such as numerical quadrature integration.

3. Update the site parameters according to (22) by damping the updates with $\delta \in (0, 1]$. For the site approximations $\tilde{t}_{\mathbf{w},i}(\mathbf{w})$ this results in the following updates:
$$\begin{aligned}
\tilde{\mathbf{T}}_{\mathbf{w},i}^{\mathrm{new}} &= (1-\delta)\tilde{\mathbf{T}}_{\mathbf{w},i} + \delta\eta^{-1}(\hat{\boldsymbol{\Sigma}}_i^{-1} - \boldsymbol{\Sigma}_{-i}^{-1}) \\
&= \tilde{\mathbf{T}}_{\mathbf{w},i} + \delta\eta^{-1}(\hat{\boldsymbol{\Sigma}}_i^{-1} - \boldsymbol{\Sigma}_i^{-1}) \qquad (25) \\
\tilde{\boldsymbol{\nu}}_{\mathbf{w},i}^{\mathrm{new}} &= (1-\delta)\tilde{\boldsymbol{\nu}}_{\mathbf{w},i} + \delta\eta^{-1}(\hat{\boldsymbol{\Sigma}}_i^{-1}\hat{\boldsymbol{\mu}}_i - \boldsymbol{\Sigma}_{-i}^{-1}\boldsymbol{\mu}_{-i}) \\
&= \tilde{\boldsymbol{\nu}}_{\mathbf{w},i} + \delta\eta^{-1}(\hat{\boldsymbol{\Sigma}}_i^{-1}\hat{\boldsymbol{\mu}}_i - \boldsymbol{\Sigma}_i^{-1}\boldsymbol{\mu}_i). \qquad (26)
\end{aligned}$$
The site parameters $\tilde{\boldsymbol{\nu}}_{\boldsymbol{\theta},i}$ and $\tilde{\mathbf{T}}_{\boldsymbol{\theta},i}$ are updated similarly using the marginal tilted moments $\hat{\boldsymbol{\mu}}_{\boldsymbol{\phi},i}$ and $\hat{\boldsymbol{\Sigma}}_{\boldsymbol{\phi},i}$.

4. If sequential EP is used, apply rank-one posterior updates on $q(\mathbf{w}) = \mathcal{N}(\boldsymbol{\mu}_{\mathbf{w}}, \boldsymbol{\Sigma}_{\mathbf{w}})$ and $q(\boldsymbol{\theta}) = \mathcal{N}(\boldsymbol{\mu}_{\boldsymbol{\theta}}, \boldsymbol{\Sigma}_{\boldsymbol{\theta}})$.

If parallel EP is used, the posterior approximations $q(\mathbf{w})$ and $q(\boldsymbol{\theta})$ are refreshed after each sweep over the sites. In practice we first conduct few sweeps only for the likelihood sites $i = 1, \ldots, n$ to get a good data fit. Then we iterate few sweeps only for the prior sites $i = m+1, \ldots, n+m$. Finally we alternate between likelihood and prior site sweeps until convergence. Practical tips for implementing the EP updates in numerically stable manner can be found, e.g., in the appendices of [3].

References

[1] Michael R Andersen, Ole Winther, and Lars K Hansen. Bayesian inference for structured spike and slab priors. In Z. Ghahramani, M. Welling, C. Cortes, N.D. Lawrence, and K.Q. Weinberger, editors, *Advances in Neural Information Processing Systems 27*, pages 1745–1753. Curran Associates, Inc., 2014.

[2] Marcel V. Gerven, Botond Cseke, Robert Oostenveld, and Tom Heskes. Bayesian Source Localization with the Multivariate Laplace Prior. In Y. Bengio, D. Schuurmans, J. Lafferty, C. K. I. Williams, and A. Culotta, editors, *Advances in Neural Information Processing Systems 22*, pages 1901–1909. Curran Associates, Inc., 2009.

[3] Pasi Jylänki, Aapo Nummenmaa, and Aki Vehtari. Expectation Propagation for Neural Networks with Sparsity-promoting Priors, March 2013. URL http://arxiv.org/abs/1303.6938.

[4] Jose Miguel Hernández-Lobato, Daniel Hernández-Lobato, and Alberto Suárez. Network-based sparse Bayesian classification. *Pattern Recognition*, 44(4):886–900, April 2011. ISSN 00313203. doi: 10.1016/j.patcog.2010.10.016. URL http://dx.doi.org/10.1016/j.patcog.2010.10.016.

[5] T. Minka. Power EP. Technical Report MSR-TR-2004-149, Microsoft Research, 2004.

[6] Thomas Minka. Divergence Measures and Message Passing. Technical report, 2005. URL http://citeseerx.ist.psu.edu/viewdoc/summary?doi=10.1.1.314.3445.

[7] Thomas P. Minka. Expectation Propagation for Approximate Bayesian Inference. In *Proceedings of the 17th Conference in Uncertainty in Artificial Intelligence*, UAI '01, pages 362–369, San Francisco, CA, USA, 2001. Morgan Kaufmann Publishers Inc. ISBN 1-55860-800-1. URL http://portal.acm.org/citation.cfm?id=647235.720257.

[8] Carl E. Rasmussen. *Gaussian processes for machine learning*. Adaptive Computation and Machine Learning. MIT Press, 2006. URL http://citeseerx.ist.psu.edu/viewdoc/summary?doi=10.1.1.86.3414.

[9] Jaakko Riihimäki, Pasi Jylänki, and Aki Vehtari. Nested Expectation Propagation for Gaussian Process Classification with a Multinomial Probit Likelihood, July 2012. URL http://arxiv.org/abs/1207.3649.
